# Tactile Modulation of Whisking via the Brainstem Loop: Statechart Modeling and Experimental Validation

**DOI:** 10.1371/journal.pone.0079831

**Published:** 2013-11-27

**Authors:** Dana Sherman, Tess Oram, Dudi Deutsch, Goren Gordon, Ehud Ahissar, David Harel

**Affiliations:** 1 Department of Neurobiology, Weizmann Institute of Science, Rehovot, Israel; 2 Department of Computer Science and Applied Mathematics, Weizmann Institute of Science, Rehovot, Israel; McGill University, Canada

## Abstract

Rats repeatedly sweep their facial whiskers back and forth in order to explore their environment. Such explorative whisking appears to be driven by central pattern generators (CPGs) that operate independently of direct sensory feedback. Nevertheless, whisking can be modulated by sensory feedback, and it has been hypothesized that some of this modulation already occurs within the brainstem. However, the interaction between sensory feedback and CPG activity is poorly understood. Using the visual language of statecharts, a dynamic, bottom-up computerized model of the brainstem loop of the whisking system was built in order to investigate the interaction between sensory feedback and CPG activity during whisking behavior. As a benchmark, we used a previously quantified closed-loop phenomenon of the whisking system, touched-induced pump (TIP), which is thought to be mediated by the brainstem loop. First, we showed that TIPs depend on sensory feedback, by comparing TIP occurrence in intact rats with that in rats whose sensory nerve was experimentally cut. We then inspected several possible feedback mechanisms of TIPs using our model. The model ruled out all hypothesized mechanisms but one, which adequately simulated the corresponding motion observed in the rat. Results of the simulations suggest that TIPs are generated via sensory feedback that activates extrinsic retractor muscles in the mystacial pad. The model further predicted that in addition to the touching whisker, all whiskers found on the same side of the snout should exhibit a TIP. We present experimental results that confirm the predicted movements in behaving rats, establishing the validity of the hypothesized interaction between sensory feedback and CPG activity we suggest here for the generation of TIPs in the whisking system.

## Introduction

Many behaviors in which animals move their sensors in a repetitive, stereotyped pattern, such as whisking, sniffing, tasting, and looking, are based on an active, often periodic sampling of the environment by the sensor organs [1]–[9]. Periodic sampling is a class of closed-loop motor-sensory interactions that is hypothesized to be driven by the interaction of central pattern generator (CPG) activity and sensory feedback. However, the nature of the modulation of CPG activity by sensory signals, and the dependency of motor patterns on sensor-environment interactions are not yet understood. Additionally, it is unclear where in the brain these interactions occur. In this study, we used the well-studied brainstem loop of the vibrissal system of rats and a well-characterized vibrissal movement, “touch-induced pump” (TIP), to explore the interactions between CPG activity and sensory feedback.

A whisking CPG(s) is thought to dominate the control of “free-air exploratory whisking”, a perceptual behavior in which the vibrissae are swept back and forth rhythmically [1], [2], [4], [6]–[8] and in a coordinated manner [6], [10], [11]. The existence of a CPG was demonstrated by observations that showed that whisking in adult rats continues in the absence of peripheral sensory inputs [4], [12], [13] and of descending (e.g. cortical) control mechanisms [14], [15]. Such a CPG was shown to reside within the brainstem [16], [17]. The existence of whisking motor modulation based on whisker-derived sensory feedback was demonstrated behaviorally [11], [18]; however, no explicit mechanism has been suggested so far to account for this modulation. We herein present such a mechanism for the induction of TIPs.

TIP is a whisking behavior that occurs upon whisker-object contact, and consists of a rapid retraction and protraction of the whiskers, during which contact with the object is preserved [4], [11]. The TIP was chosen as a benchmark for theoretical modeling of sensory modulation of CPG-driven behavior, as it is sufficiently quantified and hypothesized to convey sensory feedback via brainstem circuitry.

We combined experimental observations with a dynamic, bottom-up computerized model, which we built using the visual language of statecharts [19] to investigate the role of the CPG, sensory feedback, and the interactions between them in controlling touch-induced vibrissae motion. Several biological systems have proven to be highly suitable for statechart-based modeling [20]–[23]. Here we present the first statechart model of a neural system. Using statecharts, we modeled the brainstem loop of the whisking system, including the whisker-follicle complex, the primary afferents, the trigeminal sensory nuclei, and the facial nucleus. The main advantage of such a statechart model is its object-based modeling approach, which allows a simple and unbiased analysis of the studied system (see Modeling approach and Model behavior in statecharts, under Materials and Methods). In this model, periodic whisking is assumed to be induced by a tri-phasic CPG (see Materials and Methods). As our interest was in low-level sensory modulations of CPG activity, top-down modulations of the CPG and of the brainstem loop are not modeled.

Our model provides a possible mechanism for TIP generation, solely dependent upon sensory modulation of CPG activity at the level of the brainstem. Furthermore, the model provided several predictions regarding touch-induced vibrissae movements, for which we herein provide empirical validation. In a wider context, our results can be generalized to help in the understanding of CPG-sensory feedback interactions in any adaptive, periodic behavior.

## Materials and Methods

### The model

#### Modeling approach

This study was performed using a computerized model implemented in the visual language of statecharts [19], using the Rhapsody tool [24]. This language provides the means required to describe the complex behavior of a system in an understandable and unbiased form [25]. Using a bottom-up approach, the behavior of the system is defined through the behavior of its various components. The relatively simple behaviors of individual components are described separately. These are then assembled in a dynamic fashion, without regard to how they will work in the assembly, to give rise to more complex, higher-level behaviors. Thus we get non-predefined, dynamic, complex behaviors of the system, which can be analyzed.

#### The modeling and analysis tools: Statecharts, Rhapsody, Matlab and Visual Studio

Using the object-oriented variant of the visual language of statecharts [19] described in [24], a state-based hierarchical and concurrent transition diagram (statechart) was generated to graphically describe the discrete behavior of each component of the modeled brainstem loop, via the use of (1) states, (2) events that cause transitions between states, and (3) actions that generate events (and are transmitted from one component to another). In statecharts, states can be nested inside other states, creating sub-states, which enables description at multiple levels. States may also be divided into orthogonal states, thus modeling concurrency, and allowing each component to reside simultaneously in several independent states.

Using the Rhapsody tool [24], the behavior defined by the various statecharts was executed collectively, giving rise to a dynamic simulation that allowed interactions between the different objects (via actions), responded to events and changed objects' states and parameters during run time.

During model execution, a server-client interface was used in order to allow the model (written in Java code) to frequently use a Matlab function that transformed motor neurons' firing rate to whisker motion (see Muscle forces & whisker motion in File S1).

Analysis of the dynamics of the resulting integrated behavior was done using a Visual Studio application that provided a 2D animation to help visualize vibrissae motion.

#### Model specifications

The model consisted of six types of elements that participate in composing the brainstem loop:

1. Neurons:
  1. Primary afferents (SN1s), which directly innervate the whisker follicles and convey raw sensory input to the brain. SN1 cell bodies are located in the trigeminal ganglion (TG)

The SN1s in the model were divided into four subgroups, according to the type of sensory input they relayed: whisking (SN1_W), contact (SN1_C), pressure (SN1_P) and detach (SN1_D) cells [26].

  1. Secondary afferents (SN2s), which receive synaptic input from SN1s and project to the motor neurons. SN2 cell bodies are located in the trigeminal nuclei (TN)

In the model, each SN2 was innervated by exactly one type of SN1: SN1_W, SN1_C, SN1_P or SN1_D. Thus, SN2s were also divided into four subgroups, according to the type of sensory input they relayed: whisking (SN2_W), contact (SN2_C), pressure (SN2_P) or detach (SN2_D) [26], [27].

  1. Efferent motor neurons (MNs), which receive synaptic inputs from SN2 cells and project to mystacial pad muscles. MN cell bodies are located in the lateral facial nucleus (FN)

The MNs in the model were divided into three subgroups, according to the type of muscle they innervated: intrinsic (Int_MN), extrinsic protractor (ExtP_MN), and extrinsic retractor (ExtR_MN) cells [28], [29].

1. Mystacial pad muscles, which were innervated by MNs and responded to the motor output by contraction, which moved the whiskers attached to them. This group of elements was divided into three subgroups, according to both anatomical and functional properties of the different types of mystacial pad muscles in the rat:
  1. Intrinsic muscles (Int_muscle), which were innervated by Int_MNs and whose contraction resulted in an active forward movement of the whiskers attached to them.
  2. Extrinsic protracting muscles (ExtP_muscle), which were innervated by ExtP_MNs and whose contraction resulted in an active forward movement of the whiskers attached to them, and in a forward pad translation.
  3. Extrinsic retracting muscles (ExtR_muscle), which were innervated by ExtR_MNs and whose contraction resulted in an active backward movement of the whiskers attached to them, and in a backward pad translation.
2. Whiskers, which moved in response to muscles contraction.
3. Central pattern generators (CPGs), which innervated the MNs

The model included three CPGs, each innervating one type of MNs: an intrinsic (Int_CPG), extrinsic protracting (ExtP_CPG), and extrinsic retracting (ExtR_CPG) CPG, which innervated Int_MNs, ExtP_MNs, and ExtR_MNs, respectively [30].

1. An obstacle, which, if present in the whiskers' sweeping range, induced whisker-obstacle contacts.
2. A manager, which acted as an external environment object that passed information between objects and supported technical issues. This component did not simulate any biological component directly.

#### Model assumptions

**Figure 1:**
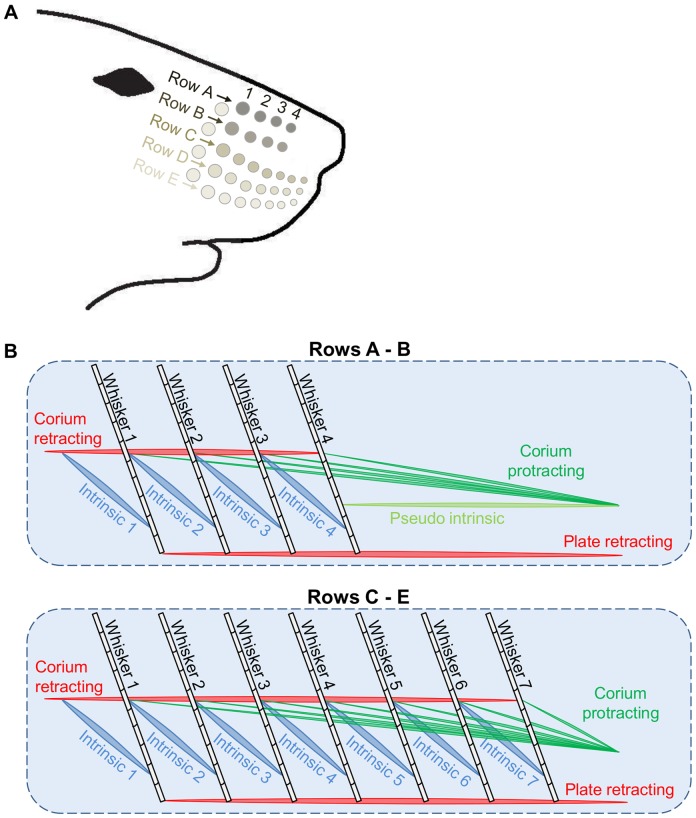
The spatial arrangement of the whiskers and muscles in the model. (**A**) Five rows of ipsilateral whiskers. (**B**) Each whisker (black) is attached to two intrinsic muscles (blue), two extrinsic retracting muscles (red), and one extrinsic protracting muscle (green). In rows A–B, the rostral intrinsic muscle of the most rostral whisker is replaced by a pseudo-intrinsic muscle (light green), whereas in rows C–E this muscle is removed. Intrinsic muscles and whiskers are indexed in an increasing order, starting at 1, from caudal to rostral.

Five rows of whiskers located at one side of the snout were modeled. Two rows were implemented as four-whisker rows, representing rows A–B in the rat, and the other three as seven-whisker rows, representing rows C–E (Figure 1A).

In both types of rows, each whisker was attached to several muscles [31] (Figure 1B):

1. Two intrinsic muscles: one rostral and one caudal. In all rows (A–E), the most rostral whisker was attached to only one intrinsic muscle, caudally, as observed in the rat [31].
2. One superficial extrinsic protracting muscle. In rows A–B, the most rostral whisker was attached to an additional deep extrinsic protracting muscle – a pseudo-intrinsic muscle [31].
3. Two extrinsic retracting muscles: a superficial muscle and a deep muscle.

The extrinsic muscles in the model and their equivalents in the rat can be found in Table 1.

**Table 1:** The extrinsic muscles in the model.

	Rows A–B		Rows C–E
Superficial protracting	PMS		PMI
Deep protracting	PI*		-
Superficial retracting	NL + ML		
Deep retracting	PIP		MP + MS

Each row of whiskers is attached to one superficial extrinsic protracting muscle, which represents Pars media superior (PMS) or Pars media inferior (PMI) of the M. nasolabialis profundus, for rows A–B or C–E, respectively. Each of the most rostral whiskers in rows A–B is also attached to a deeper extrinsic protracting muscle, which respresents the pseudo-intrinsic (PI) portions of the Pars interna of the M. nasolabialis profundus. The PI can be activated together with the PMS, or separately (by intrinsic MNs). In addition, each row is attached to two extrinsic retracting muscle groups: superficial retracting muscles and deep retracting muscles. The superficial retracting muscles represent M. nasolabialis (NL) and M. nasolabialis superficialis (NLS) for rows A–E. The deep retracting muscles represent Pars interna profunda (PIP) of the M. nasolabialis profundus for rows A–B, and Pars maxillaris profunda (MP) and Pars maxillaris superficialis (MS) of the M. nasolabialis profundus for rows C–E.

* Attached only to whiskers A4 and B4.

**Figure 2:**
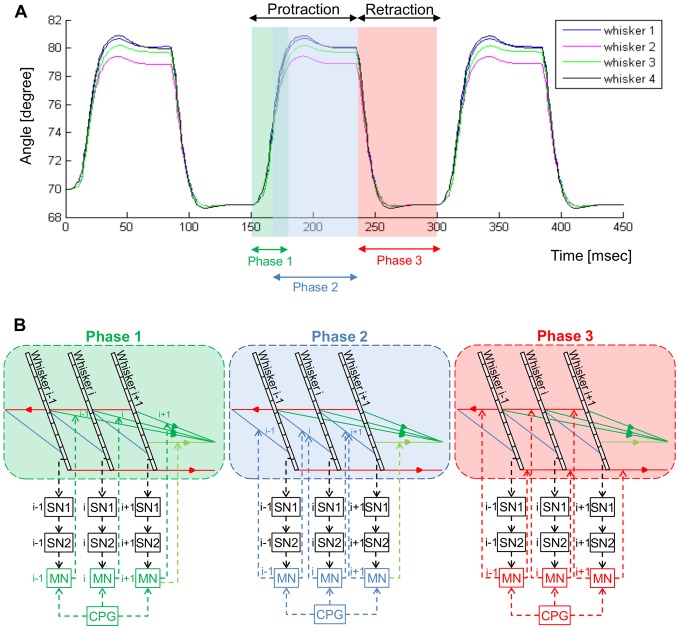
Simulated free-air whisking, induced by a tri-phasic CPG. (**A**) Whisking trajectory of a representative row of four whiskers. (**B**) The three phases of whisking are generated by a tri-phasic activation of three different groups of muscles: extrinsic protractor (Phase 1, green), intrinsic (Phase 2, blue), and extrinsic retractor (Phase 3, red) muscles. Phases 1 and 2 result in a forward whisker motion (protraction); phase 3 yields a backward motion (retraction). Note that in four-whisker rows the rostral intrinsic muscle of the most rostral whisker is replaced by a pseudo-intrinsic muscle, and that in seven-whisker rows (not shown) it does not exist. The CPG-induced tri-phasic activation may be modulated by sensory feedback.

A combined, tri-phasic activation of the above muscles simulated free-air whisking motion (Figure 2) [30]: First, extrinsic protracting muscles were activated, pulling the pad forward and initiating whisker protraction (Phase 1). Second, the intrinsic and pseudo-intrinsic muscles were activated, further moving the vibrissae forward (Phase 2). Third, relaxation of protracting muscles (both extrinsic and intrinsic) occurred, while extrinsic retracting muscles were activated, pulling the pad and the whiskers backward (Phase 3). Phases 1 and 2 gave rise to a forward whisker motion (protraction), which was followed by a backward motion (retraction), evoked by phase 3.

The timed activation of the different muscles was controlled by the coordinated firing patterns of the different CPGs, exciting the different types of MNs at the appropriate times. Each CPG stimulated all the MNs of a certain type, which together innervated all the modeled whiskers (Figure 2B). Thus, the activation of a CPG affected the motion of all the ipsilateral whiskers in a same manner. In this model, each CPG was part of an open loop and thus its behavior was not regulated. Free-air whisking motion induced by the behavior of the CPGs is further described in the “Model behavior in Statecharts” section.

**Figure 3:**
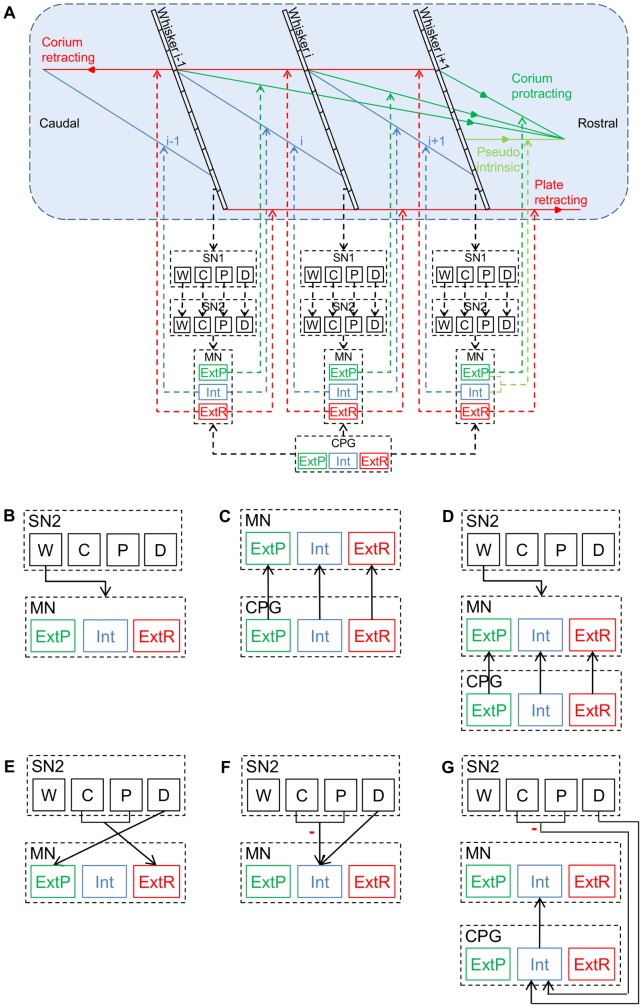
Model configurations. (**A**) In all model configurations, each whisker is exclusively innervated by one pool of SN1s, one pool of SN2s, and one pool of MNs. In each sensory feedback loop, SN1s of a certain type (i.e., whisking (W), contact (C), pressure (P) or detach (D)) innervate the corresponding type of SN2s, and MNs of a certain type (i.e., intrinsic (Int), extrinsic protractor (ExtP) or extrinsic retractor (ExtR)) innervate all the muscles of the corresponding type that are attached to the innervated whisker. Different model configurations differ by the hypothesized pre-synaptic source that innervates the MNs. (**B–D**) Pre-MN-to-MN connections in the different hypothesized whisking-inducing mechanisms: (**B**) generation of whisking by sensory feedback alone, (**C**) generation of whisking by a tri-phasic CPG, (**D**) generation of whisking by both a tri-phasic CPG and sensory feedback. (**E–G**) Pre-MN-to-MN connections in the different hypothesized TIP-inducing mechanisms: (**E**) direct excitation of retractor MNs (E–R), (**F**) direct inhibition of protractor (intrinsic) MNs (direct I-P), and (**G**) indirect inhibition of protractor (intrinsic) MNs (indirect I–P). All connections described in the figure are excitatory, except for those between SN2_C/P and their post–synaptic target in (F) and (G) (indicated by (-)).

CPG-induced MNs activity, and thus whisker motion, could be modulated by another pre-synaptic source, SN2s, which took part in a feedback loop. Separated sensorimotor feedback loops were implemented for the different whiskers, to allow each whisker to affect its own motion (Figure 2B). Feedback loops implementation is described in more detail in Figure 3 and in File S1.

Although each whisker was innervated by a separate pool of neurons, sensory feedback derived from one whisker could affect the motion of other whiskers, via muscles that connected the whiskers. The extrinsic muscles (either protracting or retracting) were assembled into groups that affected the motion of several rows of whiskers, as described in Table 1 [32]. For example, when MNs in row C activated the superficial extrinsic protracting muscle of row C (part of the PMI complex in Table 1), all three superficial extrinsic protracting muscles in rows C–E contracted, moving all whiskers in rows C–E. In addition, sensory feedback that activated the intrinsic muscles attached to the innervated whisker, affected the movement of that whisker together with its two neighboring whiskers, both the caudal and the rostral whisker (if they existed). The whiskers within a row were assumed to move within a single plane, without rotating about their own axis. For simplicity, the whiskers in the model were considered rigid.

#### Model behavior in statecharts

To illustrate a model simulation, we describe here the dynamic process of generating free-air whisking motion. In the model, whisking motion resulted from the dynamically combined behavior of four types of elements: a CPG, a MN, a muscle, and a whisker. We start by describing the behavior of each of these four elements separately, and then describe how their combined behaviors gave rise to whisking motion. Similar descriptions of other model elements can be found in File S1 (see Model behavior in statecharts) and in Figure S2.

**Figure 4:**
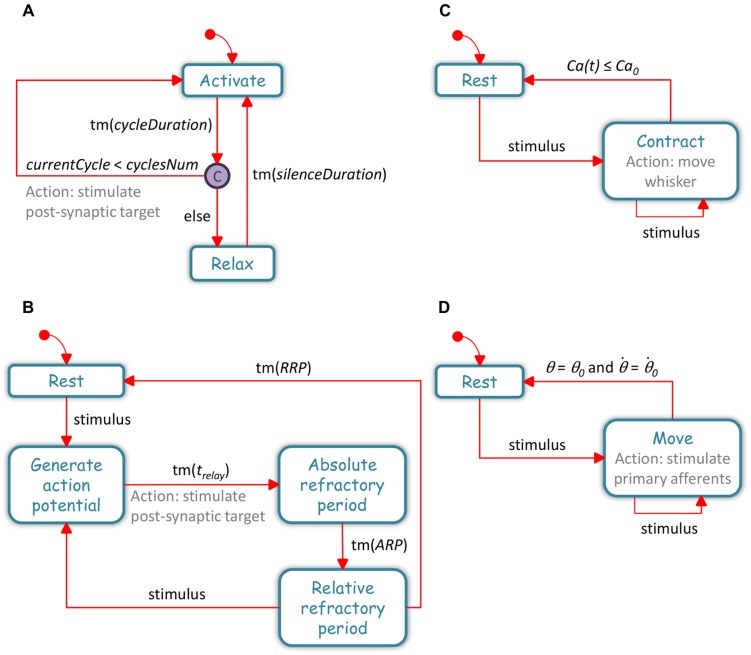
The statecharts in the model. Each statechart describes the behavior of an element of a certain type: (**A**) CPG (**B**) Neuron (**C**) Muscle (**D**) Whisker. The blue boxes indicate the states of the relevant element. The red arrows, together with the triggering events written next to them, describe the transitions between the states. Actions are indicated in gray. → points to the initial state upon model execution. © indicates a condition connector. tm(*X*) indicates a timeout of *X* msec; the values of the different *X* parameters are specified in File S1. When the model is executed, many copies of each statechart are generated, one for each of the actual components of the parent element. As the simulation advances, each component responds to various events by changing its states and parameter values accordingly, and by transmitting events to itself/other components.

The CPG statechart, whose transition diagram is shown in Figure 4A, describes the behavior of a CPG. The CPG has two states, and at any moment it can be in exactly one state: (1) “Activate” – a stimulation period, during which the CPG stimulates all MNs of the corresponding type simultaneously, and at a constant rate, and (2) “Relax” – a “silent” period, during which no stimuli are sent to the MNs. The CPG can move between states following the occurrence of a triggering event specified next to the transition arrow. In the model, the CPG stays several tens of milliseconds in each state, and continuously switches between the two states upon the occurrence of a timeout (tm) trigger.

The MN statechart has four states (Figure 4B): (1) “Rest” – in which the MN is inactive, (2) “Generate action potential” (generateAP) – in which the MN prepares to evoke an action potential (AP) and to transmit the signal to its post-synaptic muscle/s, (3) “Absolute refractory period” (ARP) – in which the MN is inactive, but in comparison to the “Rest” state, it cannot respond to any stimulus it receives. Thus, when being in this state the cell can never evoke an AP, (4) “Relative refractory period” (RRP) – which is very similar to the “Rest” state, during which the MN is inactive, but from which it can become activated. The difference between this state and the “Rest” state is that here the *threshold* is higher; i.e., the stimulus should be stronger.

The muscle statechart has two states (Figure 4C): (1) “Rest” – in which the muscle is relaxed, and (2) “Contract” – in which it contracts.

The whisker statechart also has two states (Figure 4D): (1) “Rest” – in which the whisker does not move, and (2) “Move” – in which the whisker moves.

Since statecharts are fully executable, one can capture the dynamics of the modeled behaviors by executing the model. When the simulation is initiated, many components are created for each element, with each component receiving its own copy of its parent's initialized statechart and initialized set of parameters, which together describe the component's independent behavior. The number of components in the model and a full list of model parameters are specified in Figure S1 and in File S1. The simultaneous execution of the statecharts of all the model's components resulted in a combined dynamic behavior of the system, as follow: Upon model execution, three copies of the CPG statechart were generated, one of each *type* (a CPG parameter): extrinsic protractor (ExtP_CPG), intrinsic (Int_CPG), and extrinsic retractor (ExtR_CPG). Each CPG started in the “Activate” state and its *currentCycle* parameter was assigned to zero. After a few milliseconds (*cycleDuration*), the CPG exited the “Activate” state and reached a condition connector, from which it could move to either the “Activate” state or the “Relax” state, depending on the fulfillment of a specific condition: If the value of *currentCycle* was lower than the constant *cyclesNum*, another parameter of the CPG, the CPG transmitted a stimulation event to all of its MNs (of the corresponding *type*) and re-entered the “Activate” state (and incremented its *currentCycle*), otherwise it moved to the “Relax” state. The condition held for several iterations, keeping the CPG in the “Activate” state for several tens of milliseconds. When the condition was no longer valid, the CPG moved to the “Relax” state. After a tm of several tens of milliseconds (*silenceDuration*) the CPG returned to the “Activate” state (and reset its *currentCycle*).

While in the “Activate” state, each MN innervated by the CPG received successive stimulation events. Upon the first stimulation, the MN exited the initial “Rest” state and entered the “GenerateAP” state in which it prepared to fire. After a few milliseconds (*t_relay_*), the MN exited the state, transmitted a stimulation event to its post-synaptic target/s, and entered the “ARP” state. This period also lasted several milliseconds (*ARP*), during which the MN could not respond to any stimulus. Next, the cell entered the “RRP” state and stayed there for a few tens of milliseconds (*RRP*). If stimulated during the RRP period, it moved to the “GenerateAP” state, and otherwise, it returned to “Rest”.

Many MNs of a certain *type* innervated a single muscle of the corresponding *type*. Upon the first stimulation event transmitted to the muscle, the muscle exited the initial “Rest” state and entered the “Contract” state. The stimulus affected several muscle parameters, including its cytoplasmic Ca^+2^ concentration (see Muscle's *Ca(t)* in File S1). The muscle stayed in the “Contract” state until its *Ca(t)* decreased below a certain threshold (*Ca_0_*). If while in the “Contract” state the muscle received another stimulus, it re-entered the “Contract” state with the updated parameter values.

Every time the muscle entered the “Contract” state it transmitted a move event to its whisker(s). Every whisker can receive a move event from several muscles, and upon the first stimulation event, the whisker exited its initial “Rest” state and entered the “Move” state, in which it stayed until its angle and angular velocity returned to their resting values (i.e., 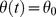 and 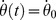). The angle and angular velocity were constantly updated each time a muscle that was attached to the whisker transmitted a move event. The muscles' force magnitude (*A*) and the resulting whisker motion were calculated using a Matlab function (see Muscle forces & whisker motion in File S1).

The three *type*s of CPGs were activated during different time periods along the whisk cycle (see CPGs' active period in File S1), resulting in a timed activation of different types of MNs and muscles, which gave rise to the free-air whisking motion.

The complexity of the dynamic behavior of the system described here makes it very suitable for statechart modeling. This complexity stems from highly concurrent and time-intensive changes of the system, due to constant interactions of the (hundreds of) system's components with each other. Such complex behavior can be analyzed when modeled by statecharts, using the kinds of execution and analysis techniques offered by the Rhapsody tool [24]. The resulting dynamic behavior of the system can be quantified via the emerging whisker motion. Comparing the simulated whisker motion to that observed in the rat allows testing the model.

#### Model configurations

In this study, three hypothesized whisking-inducing mechanisms were inspected, differing from each other in the pre-synaptic sources that innervated the MNs during free-air whisking (Figure 3B–D). Each type of MNs was innervated by: (1) a CPG of the corresponding type, (2) by SN2_Ws, or (3) by both a CPG of the corresponding type and SN2_Ws. Free-air whisking described in the last section, “Model behavior in statcharts”, was induced by mechanism (1).

In all three configurations, the cumulative stimulus that a given MN received had to cross a *threshold* in order for the MN to successfully fire. In model configuration (1) or (2), stimuli by the CPG or SN2s alone (respectively) allowed the MNs to cross *threshold* and fire. In contrast, in model configuration (3), only a mutual stimulation by both SN2s and the CPG allowed the MNs to cross the *threshold* and successfully fire. The tuning of synaptic strengths required for each configuration is assumed to be accomplished during development by behaviorally-controlled local learning rules [33]–[36].

Independent of the hypothesized whisking-inducing mechanism, this study also examined three hypothesized neural mechanisms for TIP-generation (Figure 3E–G): (1) “E-R”, excitation of retractor MNs (ExtR_MN), directly induced by sensory feedback, (2) “direct I-P”, inhibition of protractor MNs (Int_MN), directly induced by sensory feedback, and (3) “indirect I-P”, inhibition of protractor MNs (Int_MN), indirectly induced by sensory feedback.

### Animals

Whisker movements obtained by the model were compared to those measured in head-fixed rats by Deutsch et al. [11], and to whisker movements measured here in freely-moving animals. In the following sections we describe the methods used in this study for collecting data from behaving rats.

The whisking patterns of albino Wistar rats (n = 3) were measured. All whiskers were trimmed, except for one row of whiskers (C) on each side of the snout. This configuration was chosen in order to simplify the tracking of the whiskers in post-processing (see below). Trimmed whiskers were clipped close (∼1 mm) to the skin during Dormitor anesthesia (0.05 ml/100 g, S.C.) the day prior to behavioral recording. Recordings were performed prior to and following transectioning of the infraorbital branch of the trigeminal maxillary nerve (IoN).

### Ethics statement

Animal maintenance and all experimental procedures were conducted in accordance with the guidelines of the National Institutes of Health (USA) and The Weizmann Institute of Science. The protocol was approved by the Council for Experiments on Animals of the Weizmann Institute of Science (Application Number: 01260212-2). Surgeries were performed under medetomidine hydrocholoride and ketamine anesthesia. Efforts were made to minimize suffering, including analgesiac injections post-operatively (Rimadyl). After the experiment was completed, animals were sacrificed using an overdose of barbituates (Pentobarbital).

### Transectioning of the IoN

Before surgery, rats were removed from their cage and anesthetized (medetomidine hydrocholoride, 0.05 ml/100 g, S.C.; ketamine 79 mg/kg, S.C.). The rats were then mounted in a stereotaxic device. An incision was made caudal to the whisking pad. The IoN was exposed where it emerges from the eye socket. A 2 mm section of the nerve was cut out in order to prevent nerve regeneration. The wound was then sutured. Post-operatively, rats were given antibiotics (penicillin and streptomycin; Pen-Strep, 2 ml/kg, sc), analgesiac (Rimadyl, 5 mg/kg, SC in 1 ml saline), and ad libitum food and water.

### Experimental apparatus

**Figure 5:**
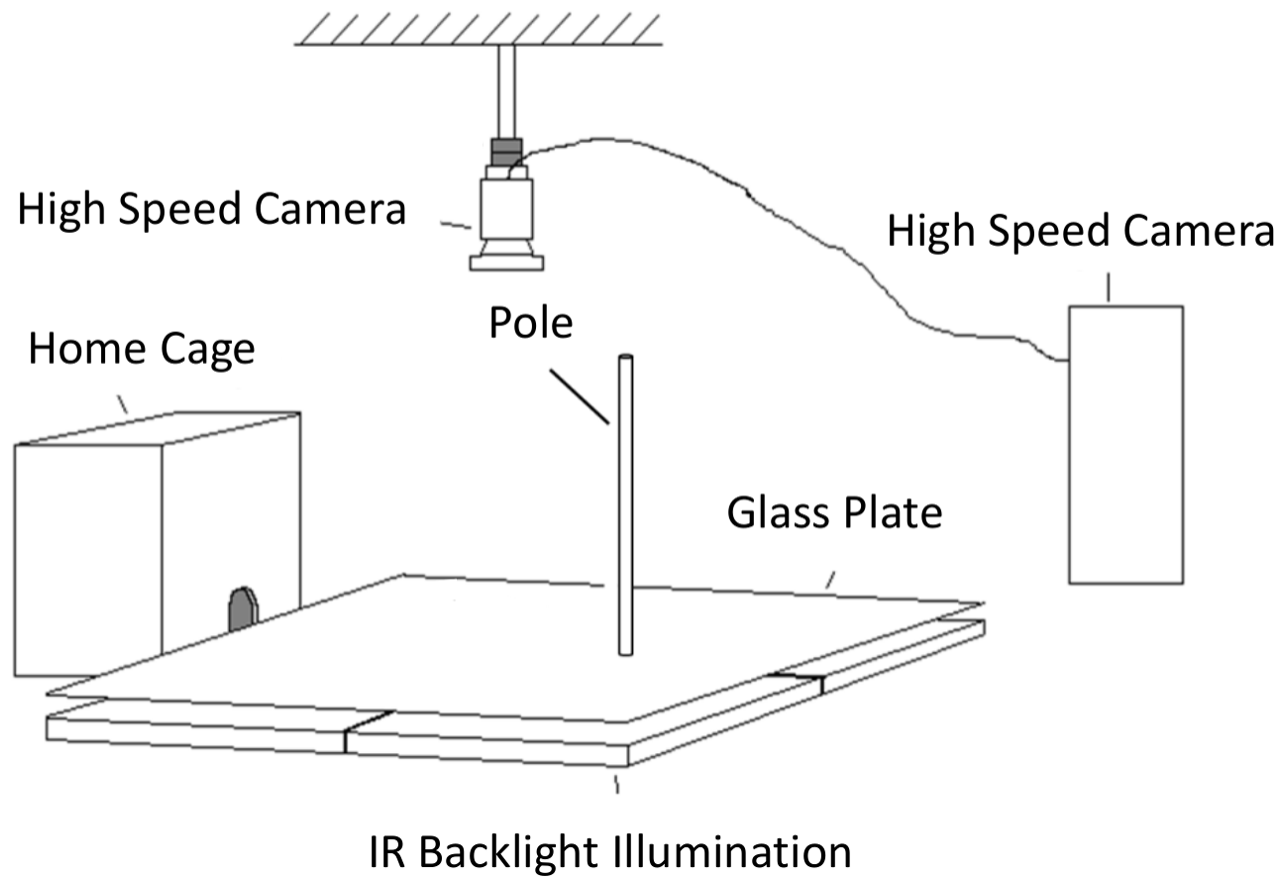
The set-up used in the behavioral experiment. The experimental procedure of the head-fixed rats is described in Deutsch et al. [11].

Behavioral experiments was performed in a darkened, quiet room. The behavioral apparatus consisted of a holding cage (25 cm width, 35 cm length, 29.5 cm height), with a small door (6.9 cm height, 6 cm width), through which the rats could emerge into the experimental area (Figure 5). Both the holding cage and the experimental area were held approximately 15 cm above the surface of a table. The experimental area consisted of a glass plate with 1–2 objects (metal poles or plexiglass cubes and cylinders) placed on the plexiglass. The location of the objects was changed between experiments, and the two obstacles were far enough from each other to prevent simultaneous touch of both obstacles. The glass plate was back-lit by an IR-lamp (880 nm wavelength, 23 cm×23 cm, Metaphase, USA). The experimental area was filmed from above by a high-speed, high-resolution camera (1280×1024 pix, 500 fps, CL600×2, Optronics, East Musckogee, OK). An in-house program, written by Dr. Enrico Segre, triggered the high-speed camera whenever the rat emerged from the holding cage into the experimental area. Video recording stopped when the rat returned to the holding cage.

### Behavioral task

An experimental session consisted of recording an animal's whisking behavior during an emergence task, over a period of 30–120 minutes. Preceding a behavioral session, the animal was removed from its cage and placed in the experimental holding cage for 15 minutes. During the acclimation period, the door in the holding cage was blocked. The experimental period began with unblocking the door in the holding cage to allow the animal to leave the holding cage and explore the experimental area at will. The experimental period varied, depending on the animal's behavior and the amount of recorded video. The experimenter's interference and contact with the animal was minimized during the experimental session and there was no interference or contact while the animal was in the experimental area.

### Video analysis

Video analysis was performed using the BIOTACT Whisker Tracking Tool [http://bwtt.sourceforge.net; 37]. This tracker provides the head position and head angle in each video frame through calculations based on the location of the rat's nose tip and the center of its snout. Additionally, the tracker identifies the rats' whiskers and provides the base angle of each of the tracker-identified whiskers with respect to the mid-saggital plane of the rat. The number of tracked whiskers varied between sessions due to differences in the whiskers' length and changes in focus owing to rat's movement.

### TIP analysis

Whisker movements were inspected both qualitatively (by eye) and quantitatively, by a computerized whisker tracker, in order to identify whiskers that “pumped” in response to touch. The identity of the touching whisker(s) varied between the different TIP events.

1. Qualitative inspection: TIP events and the identity of the “TIPing” whiskers were identified manually, by three independent observers. Since most whiskers were trimmed, individual whiskers were easily identified. Only TIPs that were detected by at least two observers were counted. The observers were in agreement on the occurrence of TIPs and the identity of the “pumping” whiskers in 80% (27 of 34) of all counted touch events.
2. Quantitative inspection: All manually counted TIPs were also analyzed using the Matlab-based WhiskerTracker image processing software [37]. This allowed a better inspection of individual whiskers' movement for determining the occurrence of TIPs.

## Results

### (1) Sensory feedback is required for TIPs generation in naturally behaving rats

Sensory feedback was indicated to be necessary for TIP generation in head-fixed rats [11]. Here we verify in freely moving rats that TIPs indeed depend on sensory feedback, by examining TIP occurrence in behaving rats whose infraorbital branch of the trigeminal maxillary nerve (IoN) was cut bilaterally. We postulated that in IoN-transected rats, where transmission of sensory information from the vibrissae to the brain was eliminated, TIPs would be absent if sensory feedback is indeed required.

Rats in which all whiskers were trimmed besides row C (bilaterally) were filmed using a high-speed video camera. Twelve movies with durations of 0.67–4 minutes were acquired before (seven) and after (five) bilateral IoN transection. In contrast to a TIP occurrence of 27% (34 TIPs out of 126 touch events) in response to whisker-obstacle contacts in intact rats, only 3% (2/61) of touch events yielded TIPs in IoN-cut rats (p = 0.028, one-tailed binomial test). Thus, bilateral IoN transection brings TIP occurrence rate to its spontaneous level (7% [11], p = 0.19, binomial test). Besides the significant reduction in TIP probability, IoN transection did not affect significantly characteristics of whisking in air as measured via cycle duration, protraction duration, whisking set-point or whisking amplitude (Dependent t-test for paired samples, n = 28; p = 0.161, p = 0.869, p = 0.125, p = 0.053, respectively). These results confirm that sensory feedback is required for TIP generation in naturally behaving rats while not significantly affecting parameters of whisking in air.

### (2) Establishing model credibility

Before inspecting possible control mechanisms that could modulate whisking motion in response to touch, we first established a model that mimicked the motion to be regulated, i.e., free-air whisking. In the rat, whisking is assumed to result from tri-phasic activation of mystacial pad musculature [30]. We implemented all vibrissal muscles that participate in generating free-air whisking [32]: extrinsic protracting, intrinsic, and extrinsic retracting muscles (see Model specifications under Materials and Methods). The sequential contraction of these three types of muscles, timed based on Fisher et al. [25], resulted in a periodic motion of all mystacial pad whiskers back and forth in a sweeping motion (Figure 2). Simulated movements obtained by the model were qualitatively and quantitatively compared with corresponding motions observed in behaving rats in order to fine-tune the mimicry of exploratory whisking motion.

#### Accurate mimicry of free-air whisking motion

During free-air whisking, rats repeatedly move their whiskers along the rostral–caudal axis in order to scan their immediate environment [1], [4], [38]. The large vibrissae on each side of the rat's snout are arranged in a grid of five rows and several (4–7) arcs (Figure 1A). Although each whisker has some capability for independent movement, the whiskers on each side of the snout generally move together [10], [11]. Thus, in principle, the modeling of an individual whisker would be sufficient to describe the motion trajectories of all ipsilateral whiskers. Yet, we modeled the entire array of ipsilateral whiskers, in order to later on investigate the effect of contact made by one whisker on the motion trajectories of other whiskers located in different rows and arcs. We start by describing the motion in a single row.

**Figure 6:**
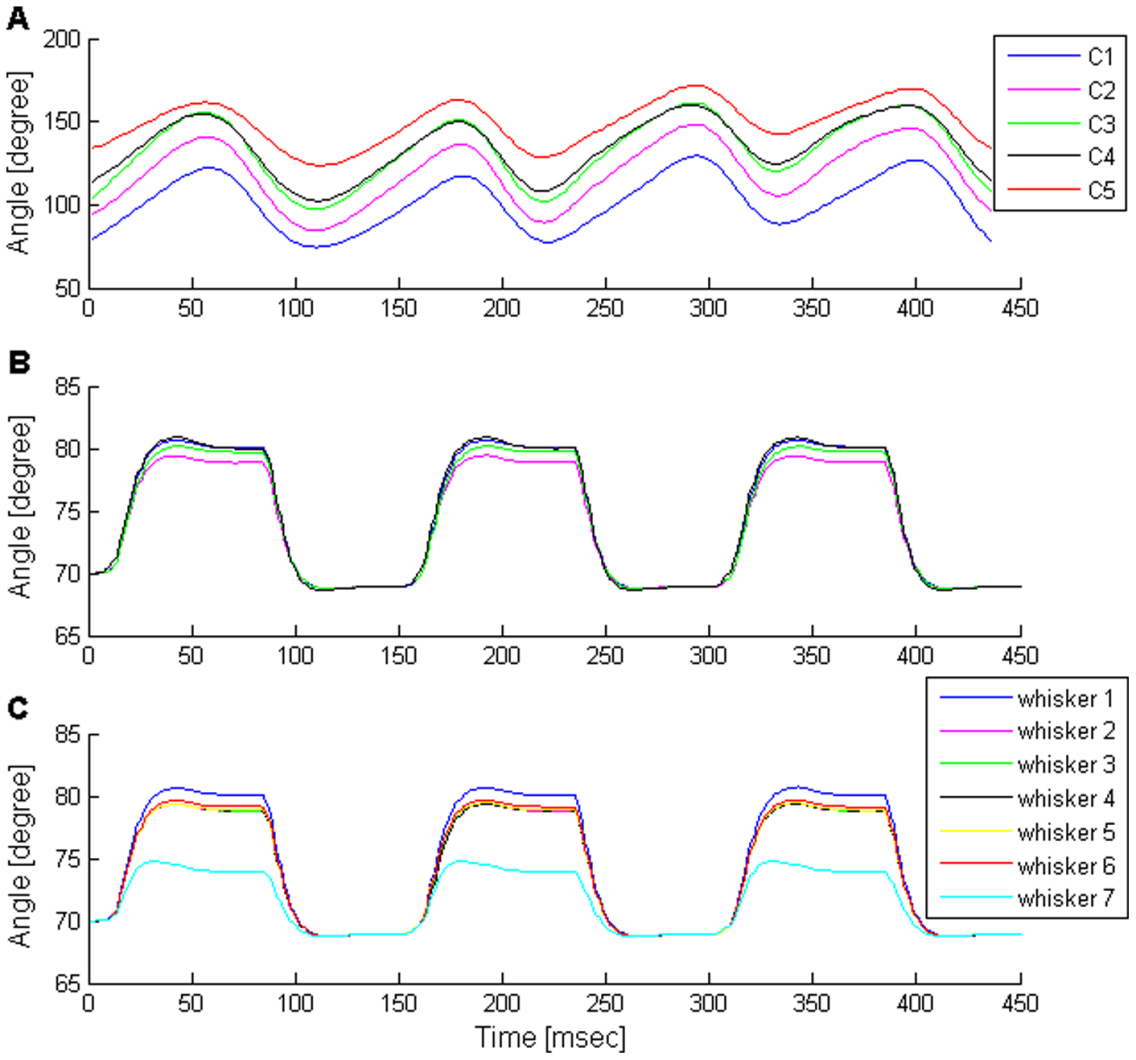
Comparison of free-air whisking between the rat and the model. (**A**) Example of free-air whisking trajectory of a single row of whiskers in a freely moving rat (filtered at 40 Hz). The tracked whiskers are numbered from caudal (C1) to rostral (C4). Whisker trajectories are highly coordinated, exhibiting a sinusoidal-like shape with an amplitude of 30–40°, and protraction and retraction phases that last about 70 and 40 msec, respectively. (**B–C**) Simulated whisking of two representative rows in the model: a four- (**B**) and a seven- (**C**) whisker rows. Whiskers are numbered from caudal (whisker 1) to rostral (whisker 7). The trajectories of all ipsilateral whiskers are highly coordinated, generating a square-wave like shape. All whiskers protract from 68.5° to 79–81° (except for the most rostral whisker in seven-whisker rows, which reaches ∼74.5°). Protraction and retraction phases last about 80 and 70 msec, respectively.

The movement of a row is characterized by homogeneous movement of all the row's whiskers [39], sweeping periodically back and forth [11] (Figure 6A). This periodic movement can be divided into whisk cycles, such that each cycle lasts 100–200 msec and is composed of a forward (protraction, 78.4±18.1 msec, mean±SD) followed by a backward (retraction, 70.3±21 msec, mean±SD) movement of the whiskers [11]. Behaving rats exhibit a whole range of whisking frequencies (mostly between 5–15 Hz [4], [12]), amplitudes (typically 10–40° [11]), and trajectories (covering the range between pure sinusoids to square waves, [e.g., fig. 2B in [10], figs. 2,9 in [12], figs. 2C,3A in [40], fig. 2 in [41]]).

Our model generated whisking trajectories that can be mapped to the low-frequency, low-amplitude, and square-wave range of the in-vivo repertoire. The movement of the five rows of whiskers found on one side of the snout was simulated. Two rows were implemented as four-whisker rows, equivalent to rows A–B in the rat, and the other three as seven-whisker rows, equivalent to rows C–E (see Model assumptions under Materials and Methods). For simplicity, a synchronized movement within the whisker array was assumed [10]. We present here whisker motion of two representative rows, since, in the absence of contacts, similar motion was obtained in rows with a similar number of whiskers. Figure 6B,C displays the angle of whiskers found in two rows as a function of time. The graph shows a coordinated movement of the whiskers while moving back and forth. In the model, a whisk cycle lasted about 150 msec, and was composed of protraction and retraction phases that lasted about 80 and 70 msec, respectively, thus mimicking biological whisking at the low end of the frequency spectrum [11]. Interestingly, the squared shape of single whisk cycles generated in these low frequencies changed to a sinusoidal shape when the whisking frequency was increased (not shown). Whisking amplitude was set to be ∼10°, at the lower end of the amplitude range observed in awake rats, in order to allow accurate calculation of whisking motion (see Muscle forces & whisker motion in File S1). From a resting angle of 70°, all whiskers reached a very similar maximal protracting angle (79–81°) except for the rostral-most whiskers in the seven-whisker rows, which only reached ∼74.5°. This reduction in protraction amplitude resulted from the model's assumption (based on anatomical data) that in a seven-whisker row, the rostral-most whisker is not attached to any protracting muscle that is active during the second phase of whisking motion (see Model assumptions under Materials and Methods). This model prediction could not be tested in our rats due to insufficient whisker length for reliable tracking.

#### Consistency with experimental data

The results described above were obtained using a neural configuration in which whisking was generated solely by the activity of a CPG. Similar results were obtained when whisking was controlled by both a CPG and sensory feedback (data not shown). In this latter configuration, CPG activity could not activate the MNs alone. Rather, the MNs required simultaneous input from both the CPG and the SN2s (see Model configurations under Materials and Methods; Note that independently of these mechanisms, three hypothesized TIP-inducing mechanisms are examined). In contrast to these two configurations, a third configuration, in which whisking was hypothesized to be generated by the activation of MNs by SN2s alone, could not produce the desired whisking pattern (data not shown). These results are consistent with experimental data that show that a CPG is necessary and sufficient to induce free-air whisking.

### (3) Model predictions

After establishing model's credibility, we examined possible control mechanisms responsible for TIPs. Deutsch et al. [11] demonstrated that, in head-fixed rats, TIPs occur due to sensory feedback which modulates whisking. They suggested that this sensory feedback occurs during the second phase of the whisk cycle (activation of the intrinsic and pseudo-intrinsic muscles), but not during the other phases. Briefly, they rejected the possibility of feedback during the first phase of whisking (activation of extrinsic protracting muscles), since this could only generate TIPs early in the whisk cycle, whereas TIPs typically occur later, near the peak of a whisking cycle. They also ruled out the possibility of feedback that modulates the backward motion generated during the third phase of whisking (activations of the extrinsic retracting muscles), since whisker velocity measured at the beginning of TIP retraction was much smaller than that at the beginning of end-of-whisk retraction, indicating that the negative velocity in the TIP is unlikely to be the beginning of the “end-of-whisk” retraction. In addition, Deutsch et al. [11] proposed that TIP-inducing sensory feedback is relayed along an anatomically-short neural loop, which would allow for the short latency of TIPs in response to touch.

Guided by these assumptions, we aimed at examining all possible sensory feedback mechanisms that could be mediated by the anatomically shortest neural loop that connects the whiskers to the mystacial muscles, the brainstem loop, in order to induce TIPs during the second phase of the whisk cycle. These mechanisms included: (1) “E-P”, excitation of protracting MNs (ExtP_MNs and/or Int_MNs), which would produce a short-term, enlarged protraction, (2) “I-P”, inhibition of the already-active second phase protracting components – Int_MNs or their pre-synaptic CPG, which would result in two discrete, non-overlapping phases of whisker protraction, or (3) “E-R”, excitation of retracting MNs (ExtR_MNs), which would interrupt the ongoing whisker protraction, and would possibly result in a reversal of movement direction. The E-P mechanism was less probable, since it would only allow producing TIPs early in protraction [11 (figure S4A)], whereas most TIPs occur in the late part of protraction, as mentioned. In contrast, the I-P and E-R mechanisms would allow generation of TIPs during the entire second phase of the whisk-cycle. These two remaining TIP-inducing mechanisms were examined here, where in the I-P mechanism we further distinguished between the two possible inhibition routes: direct inhibition of Int_MNs by trigeminal-ganglion-to-facial-nuclei inhibitory projections, “direct I-P” [42], and indirect inhibition of Int_MNs via Int_CPG inhibition, “indirect I-P”.

#### Accurate mimicry of TIPs and further characterization of this motion by model predictions

**Figure 7:**
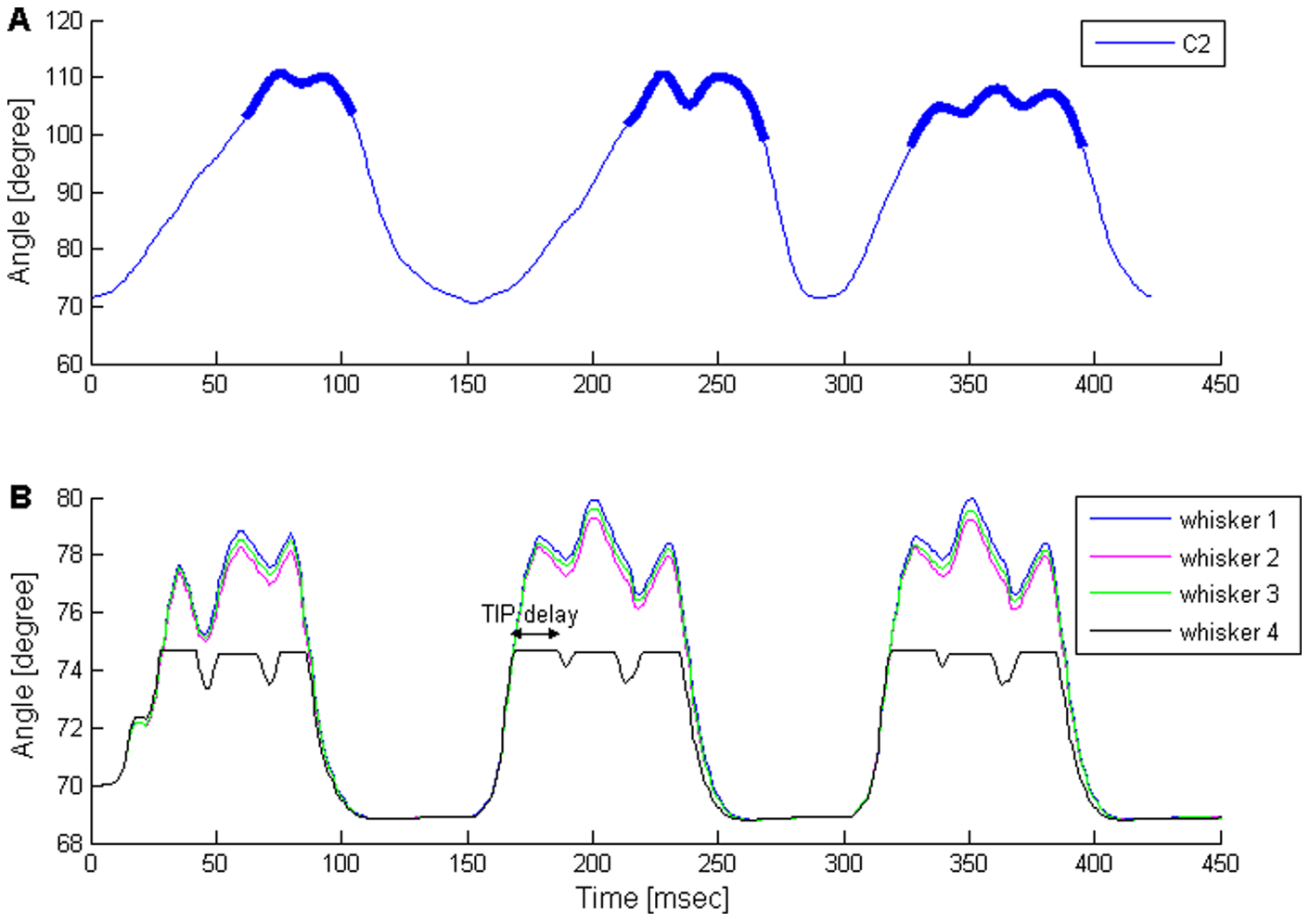
Comparison of TIPs between the rat and the model. (**A**) Example of whisking trajectory against an object of a single whisker (C2) in a head-fixed rat (filtered at 80 Hz). Whisker-object contact is indicated by bold. This example is a zoom-in of Figure 1D in [11]. (**B**) Simulated TIPs in a single row as predicted by the “excitation of retractor MNs” configuration. While only whisker 4 touches an obstacle, all row's whiskers pump. At touch onset time, the touching whisker's angle is 74.7°, and the radial distance of contact is 40% of the whisker's length. TIP delay, relative to touch onset time: 17 msec; TIP retraction amplitude: ∼1° for all the non-touching whiskers, 0.6° for the touching whisker.

Frequently, when a rat's whisker touches an obstacle it does not simply keep on protracting throughout the whole protraction phase, but, rather, “pumps” shortly (tens of milliseconds) after the beginning of touch. During the pump, the whisker briefly protracts while bending, and then a retraction of about 1° occurs, followed by another protraction (Figure 7A). During retraction, the whisker usually remains touching the object without detaching from it, by gradually straightening up [11].

A similar movement pattern was obtained by the different TIP-inducing model configurations. Figure 7B displays the angles of four whiskers found in a single row, as a function of time (similar results were obtained for seven-whisker rows, data not shown). In the figure, the touching whisker started to retract 17 msec after the beginning of touch, moving 0.6° backwards, while the other whiskers in its row retracted by about 1°. This first TIP was followed by a second TIP (see Discussion). In this example, TIPs were induced by the E-R mechanism.

As in free-air whisking, we inspected the accuracy of the simulated TIP movements obtained by the different mechanisms, by comparing the values of several quantitative characteristics of a TIP movement to the matching values measured in the rat. Our analysis only refers to the first TIP observed in each whisk cycle, since in the rat only one TIP per cycle is usually observed (see TIP occurrence, below). The results are summarized in Table 2.

**Table 2:** Comparison of TIP characteristics between the modeled TIP-inducing mechanisms and head-fixed rats.

	Model			Rat
TIP-inducing mechanism	Direct I-P	Indirect I-P	E-R	
Mean TIP delay [msec]	17–19 (Figure 8)			18
TIP amplitude of touching whiskers [°] (mean±SD)	2–2.5 (2.6±0.4)	5	0.5–0.75 (1.1±0.5)	∼1
TIP amplitude of non-touching whiskers [°] (mean±SD)	3–3.5 (3.6±1)	10*	1–2 (1.8±1.3)	1–2
“Pumping” whiskers identity	Touching whisker and its caudal and rostral neighbors (if existed)	All whiskers found on the same side of the snout as the touching whisker		All inspected whiskers found on the same side of the snout as the touching whisker**

The presented values, of both the simulations and the rat, are of TIPs induced by contacts at a radial distance of 40% from the base of the touching whisker. Values of simulated TIPs that were induced by contacts at a wide range of radial distances (20–70%) are displayed in brackets. For each TIP-inducing mechanism, values were obtained while using either one of two whisking-evoking mechanisms: generation of whisking by CPG alone or by both the CPG and sensory feedback.

* ∼10° for all non-touching whiskers in rows A–E, except for the most rostral whiskers in rows C–E, which retracted by ∼5°.

** Observed in both head-fixed and behaving rats. In head-fixed rats, TIP was observed in the two additional non-touching whiskers that where examined [11]; in behaving rats TIP was observed in all the whiskers found on the same row as the touching whisker.

#### TIP delay

**Figure 8:**
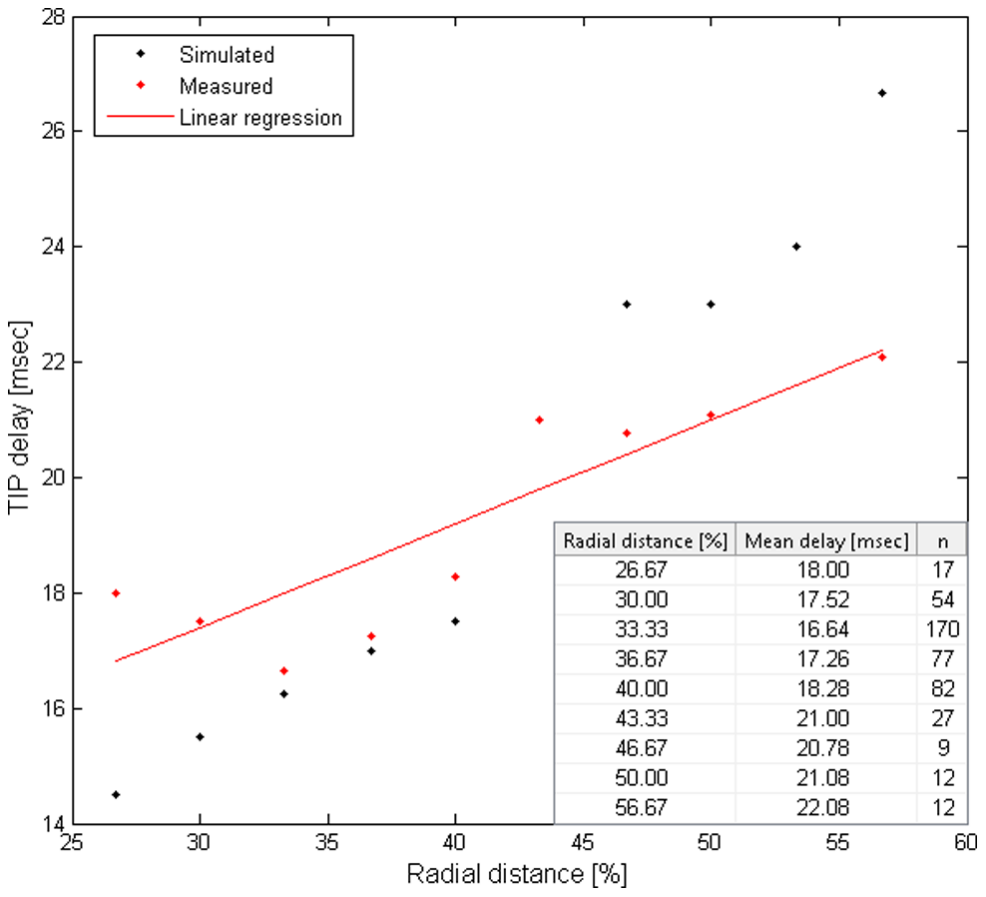
Dependency of TIP delay on radial distance of contact in head-fixed rats. Both in the model (black) and in the rat (red graph and table, p = 0.001), the mean TIP delay elongated as the radial distance from the base of the whisker increased. The radial distance was divided by the length of the touching whisker. TIP delay is relative to onset time of whisker-object contact.

In Figure 7B, the 17 msec delay in TIP onset time, relative to touch onset time, was obtained for whisker-obstacle contacts at a radial distance of 40% of the touching whisker's length (measured from the base of the whisker). This delay was obtained by all three model configurations for the specified radial distance, and its value was consistent with the mean delay observed in head-fixed rats (18.1±5.8 msec, mean±SD), in response to contact at a similar distance [11]. However, in the model, this delay varied for different radial distances of contact. All three model configurations predicted dependency between the delay in TIP onset time and the radial distance of contact: as the contact point became closer to the base of the whisker, the delay decreased (Figure 8, black). Analysis of experimental measurements from head-fixed rats revealed that such an increase in TIP delay was significant (Figure 8, red, p = 0.001). The model prediction was based on the finding that the delay in pressure cells firing (SN1_P) increases as the radial distance of contact increases [43].

TIP delays in the model were then fine-tuned to match the delays observed in head-fixed rats, which varied between 12–30 msec. This range is in agreement with experimental data, where 95% of TIPs in the rat start 12–30 msec after contact onset time.

#### TIP amplitude of the touching whisker

In contrast to the high compatibility of the delays in TIPs between all three model configurations and the rat, TIP retraction amplitude of the touching whisker obtained by only two mechanisms matched the values measured in head-fixed rats (about 1°, in response to contacts at a radial distance of 40%): E-R or direct I-P resulted in a TIP retraction amplitude of 0.5–0.75° or 2–2.5°, respectively, whereas indirect I-P yielded a too-large retraction amplitude of 5°. The difference in retraction amplitudes between the E-R vs. I-P (either direct or indirect) configurations stemmed from the different magnitudes of forces applied by the different types of muscles involved in TIP induction. In the first modeling stage, in which model's credibility was established, force magnitudes of the modeled muscles were set to certain values that yielded an accurate mimicry of free-air whisking amplitude. Accordingly, intrinsic muscles were set to apply larger force than the extrinsic retracting muscles, in agreement with experimental data [13 (figure 2B),30 (figure 7C,E)]. Hence, I-P (either direct or indirect) resulted in a larger whisker retraction than E-R. The difference in retraction amplitudes between the direct vs. indirect I-P configurations stemmed from different number of intrinsic muscles that were involved. In the direct I-P, sensory feedback derived from the touching whisker resulted in the relaxation of only two intrinsic muscles that were attached to it, whereas in the indirect I-P, sensory feedback led to the relaxation of all ipsilateral intrinsic muscles, amplifying the effect (see Model assumptions under Materials and Methods). We emphasize that neural response times to whisker detachment were similar in all three model configurations, rejecting the possibility that larger TIP retraction amplitudes were obtained due to longer lags in response to whisker detachment.

#### Spatial spread of TIP across whiskers

**Figure 9:**
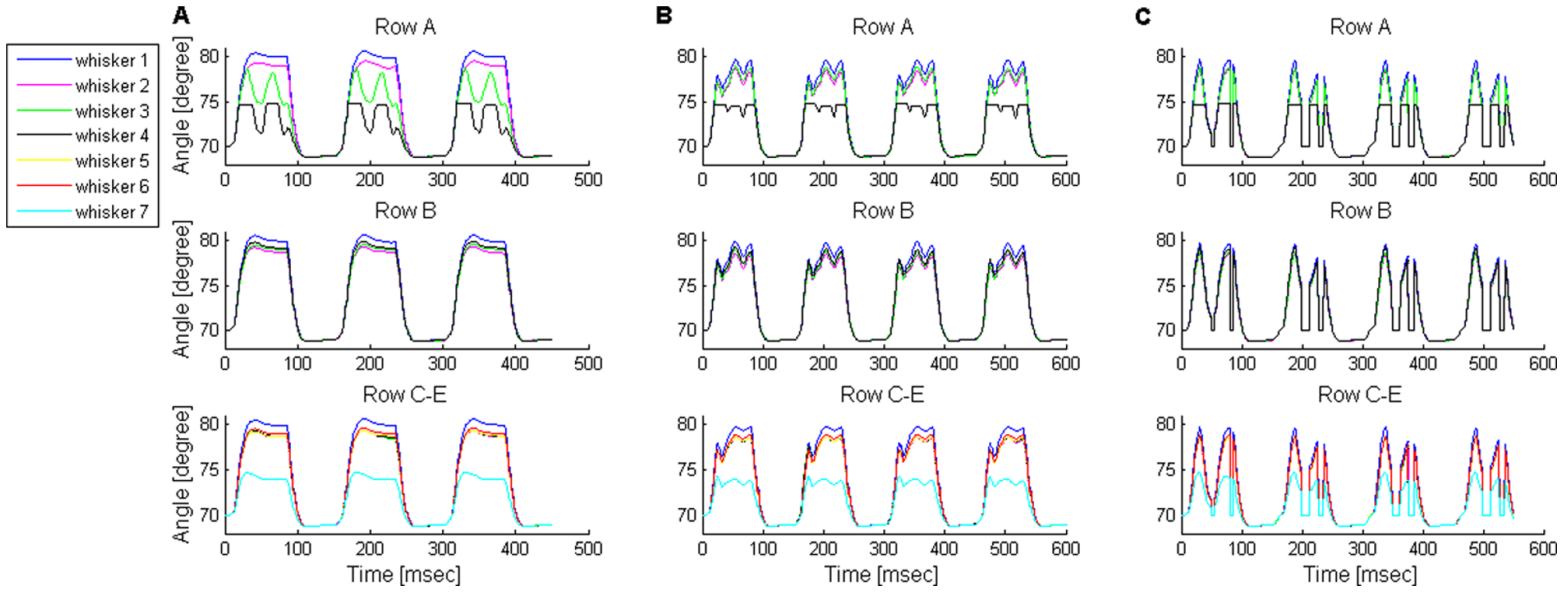
Simulated TIPs predicted by the three model configurations, following whisker A4-object contact. The angles of whiskers found in three different rows are displayed as a function of time, during TIPs. While only whisker 4 in row A touched an obstacle, all rows' whiskers pumped. Upon contact, whisker A4 angle was 74.7°; the radial distance of the obstacle was 40% of the whisker's length, measured from the base of the whisker. Since rows C–E were implemented in the exact same way, whisker motion in all three rows was identical. Therefore, whisker movement of only one representative row (row C) is displayed. (**A**) Direct I-P configuration: TIP delay of whisker A4: 19 msec; TIP retraction amplitudes: ∼3° for the non-touching whisker A3 (the touching whisker's caudal neighbor); 3° for the touching whisker. All other whiskers continue to perform unmodulated whisking motion. (**B**) E-R configuration: TIP delay of whisker A4: 17 msec; TIP retraction amplitudes: ∼2° or ∼1.5° for all non-touching whiskers in rows A–B or C–E, respectively; 0.6° for the touching whisker. (**C**) Indirect I-P configuration: TIP delay of whisker A4: 19 msec; TIP retraction amplitudes: ∼10° for all non-touching whiskers in rows A–E, besides the most rostral whiskers in rows C–E, which retracted by ∼5°; ∼5° for the touching whisker.

The different model configurations also differed in the predicted identity of the whiskers that would “pump” in response to the contact of a single whisker with an object. The direct I-P configuration predicted that in addition to the touching whisker, both of its caudal and rostral neighbors (if they existed) would also “pump” (Figure 9A). This resulted from the assumption that sensory feedback derived from the touching whisker leads to the relaxation of the two intrinsic muscles attached to it, as each muscle is also attached to one of the touching whisker's neighbors (see Model assumptions under Materials and Methods). In contrast, the other two model configurations predicted that all whiskers found on the same side of the snout would “pump” (Figure 9B,C). In the E-R configuration, the prediction resulted from the assumption that sensory feedback derived from the touching whisker excites the superficial extrinsic retracting muscles, which are attached to all ipsilateral whiskers. Alternatively, in the indirect I-P configuration, the prediction resulted from the assumption that the sensory feedback derived from the touching whisker inhibits the Int_CPG, and thus leads to the relaxation of all ipsilateral intrinsic muscles (see Model assumptions under Materials and Methods).

Following model predictions, we examined the identity of the “pumping” whiskers in behaving rats. Whisking rats in which all whiskers were trimmed except for row C (bilaterally, 5–7 whiskers on each side) were filmed using a high-speed video camera. Whisker movements were inspected both qualitatively, by three independent observers, and quantitatively, by a computerized whisker tracker, to identify TIP events and the participating “pumping” whiskers (see Materials and Methods). No rats with fully intact whisker pads were inspected, as whisker tracking and identification was not feasible with full whisker pads by any of the inspection strategies.

**Figure 10:**
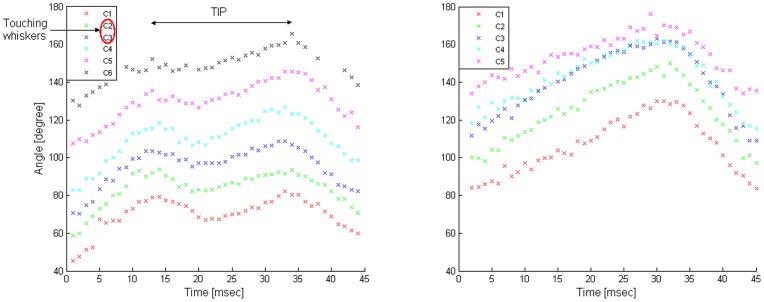
Whisker motion in a freely moving rat. The angles of the tracked whiskers (C1–C6) on the left side of the snout are displayed. More rostral whiskers were not detected by the computerized whisker tracker (see Materials and Methods). Left: TIP in all tracked whiskers, while only C2 and C3 touched an obstacle located in front of them. Right: free-air whisking, for comparison.

In 53% of the detected TIP events, all the traceable whiskers in the row “pumped” (18 of 34 studied TIPs). Out of the remaining 16 TIP events, in 11 cases 1–2 whiskers slowed down but did not exhibit detectable retraction; the other 3–5 whiskers exhibited clear TIPs. In the other five cases, 1–2 whiskers were either out of focus or too short to observe a TIP; the other 3–5 whiskers exhibited clear TIPs. The number and identity of the touching and “pumping” whisker(s), as well as the number of traceable whiskers, varied between the different TIP events, and are summarized in Table 3 (whisker identity is not specified). An example of a TIP observed in all tracked whiskers, both touching and non-touching, is displayed in Figure 10.

**Table 3:** Distribution of TIP detection in behaving rats.

Number of touching whiskers	Number of “pumping” whiskers	Number of tracked whiskers	Number of inspected events (n)	Non-pumping whiskers state^1^
1	5	6	2	1 (1 event)
		5	1	-
	4	6	2	1 (1 event) or 2 (1 event)
		5	2	1 (2 events)
		4	3	-
	3	5	1	3+4
		4	2	4 (1 event)
		3	1	-
	2	2	1	-
2	5	6	2	1 (1 event)
		5	1	-
	4	6	2	4 (1 event)
		5	3	4 (2 events)
	3	6	2	1+4 (1 event) or 2+4 (1 event)
		5	2	1+4 (1 event)
	2	4	1	4
3	3	6	1	4
	2	5	1	-
4	5	6	2	-
	3	6	1	4
		5	1	-

1 1 – out of focus; 2 – too short to tell if pump; 3 – obstructed by the object; 4 – too protracted against the object to pump.

Our results are consistent with those of Deutsch et al. [11], who tracked the simultaneous movements of three whiskers (C1, C2, and D1) bilaterally in head-fixed rats. Deutsch et al. found that in addition to TIPs in the touching whisker (C2), the other two ipsilateral whiskers usually also “pump”.

#### TIP amplitude of the non-touching whiskers

Not only did the identity of the non-touching “pumping” whiskers differed between the different model configurations, but the predicted TIP retraction amplitude of these whiskers differed as well. Following contact of a single whisker with an object at a radial distance of 40%, the E-R configuration predicted retraction amplitude of 1–2°, direct I-P predicted 3–3.5°, and the indirect I-P configuration predicted 10°. These differences resulted from similar considerations as those for the touching whiskers (see TIP amplitude of the touching whisker under Results).

Comparison of the predicted values with those measured in head-fixed rats pointed at one feedback mechanism that best fit experimental data. Deutsch et al. [11] measured a retraction amplitude of about 2° of a non-touching whisker, C1, in response to contacts at a radial distance of 40%, in accordance with the E-R configuration.

## Discussion

This study examines the roles of CPG, sensor-derived sensory feedback, and CPG-sensory feedback interactions in shaping periodic sensor movements. As a case study, we examined a well-characterized vibrissae movement that occurs following vibrissa-object contact (touch-induced pump, TIP), and which is assumed to result from the modulation of whisking-inducing CPG activity by vibrissa-derived sensory feedback. Using a dynamic, object-based computerized model built with the language of statecharts, and following Deutsch et al. [11], we examined three possible feedback mechanisms that may generate TIPs, and which pass along the brainstem loop, one excitatory and two inhibitory. Comparison of simulated and real TIP movements ruled out all hypothesized mechanisms but one: excitation of retractor motor neurons (E-R), which consistently and accurately simulated the corresponding motion observed in head-fixed rats [11] (Table 2). Moreover, several model predictions that further characterized TIP motion were empirically validated in head-fixed and freely-moving rats (Figures 8 and 10, respectively), further supporting the proposed mechanism. Previous electrophysiological studies provide additional support, as only excitatory neuronal feedback has been shown to occur in the brainstem loop in vivo [44]. These results can be generalized to help understand the nature of CPG-sensory feedback interactions in other adaptive periodic behaviors.

### Statecharts modeling

Computational models provide helpful means for understanding how behavior (e.g., whisker motion) emerges from the activity of a network of neurons, by allowing the experimenter to reproduce complex dynamic activities which can then be analyzed and explained. While both object-based and equation-based models accommodate this, the former have one major advantage in biological modeling [45]: Object-based models allow both qualitative (states) and quantitative description of biological behaviors, offering an alternative if precise quantitative biological relationships are unknown, if too many variables are involved, or if they change over time, depending on certain events. Among object-based modeling methods, the visual language of statecharts [19] provides a major practical advantage. Using the Rhapsody tool [24], statecharts is accessible through very intuitive graphical interfaces, allowing experimentalists to easily define behaviors for improving their understanding of complex biological phenomena.

### The E-R brainstem loop

The E-R mechanism is similar to the flexor (pain) reflex, where a bi-synaptic excitatory feedback activates antagonist muscles to oppose the activity of the agonist muscle [46]. The flexor reflex is usually referred to as a protection reflex whose role is to pull the body away from damaging stimuli. TIPs, in contrast, lead to a more frequent object palpation, probably for a better estimation of object's features. An interesting possibility that is raised by our results is that flexor ‘reflex’ loops may also serve perceptual functions, in cases where stimulus intensity is not painful.

Alternatively, TIPs can be considered as ‘fixational whisker movements’, as they result in the whisker centering, or ‘fixating’, on a point of interest. This can be compared with similar processes in other modalities, such as fixational eye movements in primates [47]. In both cases the scanning movements of the sensory organs involve large and small movements, where the large movements shift the scanning from one point of interest to another and the small ones explore a given point of interest. It may thus be interesting to consider an E-R like mechanism for participating in the control of fixational eye movements, and a similar principle participating in the control of sniffing [31], [48] or tasting of novel food [49].

### Model predictions

Our model provided several predictions about vibrissae movements:

1. In free-air whisking simulations, the model predicted that the most rostral whiskers in seven-whisker rows (corresponding to rows C–E in the rat) would exhibit smaller protraction amplitude (∼74.5°) than all other whiskers (79–81°) (Figure 6B,C), since no muscles were attached rostral to these whiskers [31].
2. In TIP simulations, when TIP was evoked by E-R, the model predicted a difference in TIP retraction amplitude between two groups of whiskers: those in rows A–B, and those in rows C–E. Whiskers in one group (either A–B or C–E) were hypothesized to retract by a larger extent than those in the other group, when the touching whisker was among them. This difference stemmed from the activation of deep extrinsic retracting muscles, which were separated into two different groups that were attached to either rows A–B or C–E (see Model assumptions under Materials and Methods, Table 1).

Testing these predictions is beyond the resolution of our current technology. Future inspection of these predictions by better means would allow us to further test the validity of the proposed model.

### Beyond the brainstem loop: Top-down modulations

Although our brainstem-loop model mimics within-cycle dynamics of whisker motion very well, it is limited in its ability to capture longer timescale, across-cycles dynamics. Comparison of simulated TIPs with those measured in head-fixed rats demonstrates two such dynamics. First, in head-fixed rats, TIPs induced by contacts at a radial distance of 40% occur at about 25% of the whisk cycles, such that a whisker usually “pumps” once in a whisk cycle, and more rarely twice [11]. In contrast, in our model, TIPs occurred every cycle, for all radial distances of contact, and repeated one to two times per cycle, depending on the radial distance of contact (once for radial distance >60%, twice otherwise). Second, in head-fixed rats, contact duration is doubled during whisks that exhibit TIPs in comparison to whisks with contact but no TIPs [11]. In contrast, in our model, TIP occurrence did not affect contact duration (data not shown).

We postulate that these differences stem from top-down regulation, which may modulate the magnitude and timing of brainstem-derived responses significantly [50]–[53]. More specifically, TIP probability in the rat could result from top-down regulation based on the following findings: (1) In the rat, the occurrence of TIPs was associated with a more protracted set-point [4], [11], a parameter that is believed to be controlled by higher brain areas than the brainstem – i.e., cortical [1], [54], thalamic [55] or midbrain (e.g., superior colliculus; [56]) areas; (2) TIPs are temporally clustered, occurring in successive whisks rather than distributed randomly upon touch events [11]. These findings suggest top-down gating that switches between facilitating and suppressing the excitability of sensory neurons (in the model these would be touch cells SN2_C and SN2_P), resulting in promoted and obstructed TIP periods, respectively. Further assuming that these facilitating and suppressing processes are slow processes that gradually develop over several whisk cycles, then such top-down gating could also explain the occurrence of more than one TIP per cycle: in the first facilitating cycles, the touch cells would receive relatively weak stimuli that would allow them to fire only while at “Rest”, during which their *threshold* is relatively low (see Figure 4B). These initial, weak stimuli would not allow the touch cells to cross the higher *threshold* while at “relative refractory period” state, possibly resulting in one TIP per cycle. In subsequent cycles, the stronger stimuli could allow these cells to also fire upon excitation from the “RRP” state, resulting in two TIPs per cycle, as observed by Deutsch et al. [11] and as shown in Figure 7A. The longer contact periods in the rat could result from top down regulation that resets the activity of the tri-phasic CPG in response to touch, which would elongate contact periods [16]. Thus, we speculate that integrating higher-loops into our brainstem-loop model, as well as closing the CPGs' loop, would allow for the capturing of such slow modulations.

Our bottom-up modeling approach enables us to gradually add higher loops in a simple and natural manner. In this way, increasingly complicated behaviors of the system emerge step by step, thus enabling the explanation of each incremental change. As observed in this study, extending our model may not only explain familiar behaviors, but can also reveal new, unfamiliar behaviors of the studied system. We hope that as our model evolves we will obtain more insights that will further promote our understanding of the way the vibrissal system works.

## Supporting Information

Figure S1 **The number of elements that compose a single whisker's loop.** Each whisker is innervated by a separated pool of primary afferents (SN1s), secondary afferents (SN2s) and motor efferents (MNs), which contain tens of neurons of several subtypes, as indicated in the scheme. For example, a single whisker is directly innervated by 162 SN1s which include 28 detach (D), 73 whisking (W), 33 contact (C), and 28 pressure (P) cells. Each type of SN1s innervates the corresponding type of SN2s, where a single SN2 is innervated by randomly chosen 50% SN1s of the corresponding type. Depending on the TIP-inducing configuration, different types of SN2s innervate different types of MNs (as indicated in figure 3B–G in the paper), with each MN innervated by all SN2s of the matched type. The “E-R” TIP-inducing configuration is displayed here. Each type of MNs innervates the corresponding type of muscle/s attached to the whisker (as indicated in figures 1B, 2B in the paper). In addition to this closed loop, all whiskers' MNs are innervated by the model CPGs, with each CPG innervating all MNs of the corresponding type. Note that no connections exist between sub-populations of neurons of a certain type (e.g., between whisking (SN1_W) and pressure (SN1_P) cells). * Two (instead of one) extrinsic protractor muscles are attached to the most rostral whisker in rows A–B.
(TIF)

Figure S2 **The statechart of the Obstacle element.** The behavior of the Obstacle is described in File S1.
(TIF)

File S1 **Supporting information.**
(DOCX)

## References

[1] Kleinfeld D, Berg RW, O'connor SM (1999) Anatomical loops and their electrical dynamics in relation to whisking by rat. Somatosens Mot Res 16: 69–88. 10449057 10.1080/08990229970528

[2] Knutsen PM, Ahissar E (2009) Orthogonal coding of object location. Trends Neurosci 32: 101–109. 19070909 10.1016/j.tins.2008.10.002

[3] Youngentob SL, Mozell MM, Sheehe PR, Hornung DE (1987) A Quantitative Analysis of Sniffing Strategies in Rats Performing Odor Detection Tasks. Physiology & Behavior 41: 59–69. 3685154 10.1016/0031-9384(87)90131-4

[4] Welker WI (1964) Analysis of sniffing of the albino rat. Behaviour 22: 223–244.

[5] Halpern BP (1983) Tasting and smelling as active, exploratory sensory processes. Am J Otolaryngol 4: 246–249. 6353966 10.1016/s0196-0709(83)80066-0

[6] Knutsen PM, Biess A, Ahissar E (2008) Vibrissal kinematics in 3D: tight coupling of azimuth, elevation, and torsion across different whisking modes. Neuron 59: 35–42. 18614027 10.1016/j.neuron.2008.05.013

[7] Pietr MD, Knutsen PM, Shore DI, Ahissar E, Vogel Z (2010) Cannabinoids reveal separate controls for whisking amplitude and timing in rats. J Neurophysiol 104: 2532–2542. 20844105 10.1152/jn.01039.2009

[8] Ahissar E, Knutsen PM (2008) Object localization with whiskers. Biol Cybern 98: 449–458. 18491159 10.1007/s00422-008-0214-4

[9] Land MF (2006) Eye movements and the control of actions in everyday life. Prog Retin Eye Res 25: 296–324. 16516530 10.1016/j.preteyeres.2006.01.002

[10] Bermejo R, Vyas A, Zeigler HP (2002) Topography of rodent whisking–I. Two-dimensional monitoring of whisker movements. Somatosens Mot Res 19: 341–346. 12590835 10.1080/0899022021000037809

[11] Deutsch D, Pietr M, Knutsen PM, Ahissar E, Schneidman E (2012) Fast Feedback in Active Sensing: Touch-Induced Changes to Whisker-Object Interaction. PLOS ONE 7: e44272. 23028512 10.1371/journal.pone.0044272 PMC3445569

[12] Gao P, Bermejo R, Zeigler HP (2001) Whisker Deafferentation and Rodent Whisking Patterns: Behavioral Evidence for a Central Pattern Generator. The Journal of Neuroscience 21: 5374–5380. 11438614 10.1523/JNEUROSCI.21-14-05374.2001 PMC6762837

[13] Berg RW, Kleinfeld D (2003) Rhythmic whisking by rat: retraction as well as protraction of the vibrissae is under active muscular control. J Neurophysiol 89: 104–117. 12522163 10.1152/jn.00600.2002

[14] Lovick TA (1972) The behavioural repertoire of precollicular decerebrate rats. J Physiol 226: 4P–6P. 4673550

[15] Semba K, Komisaruk BR (1984) Neural substrates of two different rhythmical vibrissal movements in the rat. Neuroscience 12: 761–774. 6472619 10.1016/0306-4522(84)90168-4

[16] Moore JD, Deschênes M, Huber D, Smear MC, Demers M, et al. (In press) A common brainstem oscillator coordinates whisking with breathing in rodents: Evidence for a master clock in orofacial behaviors.

[17] Hattox A, Li Y, Keller A (2003) Serotonin regulates rhythmic whisking. Neuron 39: 343–352. 12873389 10.1016/s0896-6273(03)00391-x

[18] Mitchinson B, Martin CJ, Grant RA, Prescott TJ (2007) Feedback control in active sensing: rat exploratory whisking is modulated by environmental contact. Proc Biol Sci 274: 1035–1041. 17331893 10.1098/rspb.2006.0347 PMC2124479

[19] Harel D (1987) Statecharts: A visual formalism for complex systems. Science of Computer Programming 8: 231–274.

[20] Harel D (2003) A Grand Challenge for Computing: Towards Full Reactive Modeling of a Multi-Cellular Animal. Bulletin of the EATCS, European Association for Theoretical Computer Science 81: 226–235.

[21] Efroni S, Harel D, Cohen IR (2007) Emergent Dynamics of Thymocyte Development and Lineage Determination. PLoS Computational Biology 3: 127–136. 10.1371/journal.pcbi.0030013 PMC1782042 17257050

[22] Setty Y, Cohen IR, Dor Y, Harel D (2008) Four-dimensional realistic modeling of pancreatic organogenesis. Proc Natl Acad Sci U S A 105: 20374–20379. 19091945 10.1073/pnas.0808725105 PMC2629264

[23] Swerdlin N, Cohen IR, Harel D (2008) The Lymph Node B Cell Immune Response: Dynamic Analysis In-Silico. Proceedings of the IEEE (special issue on Computational System Biology) 96: 1421–1443.

[24] Harel D, Gery E (1997) Executable Object Modeling with Statecharts. IEEE Computer 30: 31–42.

[25] Fisher J, Harel D, Henzinger TA (2011) Biology as reactivity. Communications of the ACM 54: 72.

[26] Szwed M, Bagdasarian K, Ahissar E (2003) Encoding of Vibrissal Active Touch. Neuron 40: 621–630. 14642284 10.1016/s0896-6273(03)00671-8

[27] Yu C, Derdikman D, Haidarliu S, Ahissar E (2006) Parallel Thalamic Pathways for Whisking and Touch Signals in the Rat. PLoS Biology 4: 819–824. 10.1371/journal.pbio.0040124 PMC1436027 16605304

[28] Klein BG, Rhoades RW (1985) Representation of Whisker Follicle Intrinsic Musculature in the Facial Motor Nucleus of the Rat. The Journal of Comparative Neurology 232: 55–69. 3973083 10.1002/cne.902320106

[29] Herfst LJ, Brecht M (2008) Whisker movements evoked by stimulation of single motor neurons in the facial nucleus of the rat. J Neurophysiol 99: 2821–2832. 18353915 10.1152/jn.01014.2007

[30] Hill DN, Bermejo R, Zeigler HP, Kleinfeld D (2008) Biomechanics of the vibrissa motor plant in rat: rhythmic whisking consists of triphasic neuromuscular activity. J Neurosci 28: 3438–3455. 18367610 10.1523/JNEUROSCI.5008-07.2008 PMC6670594

[31] Haidarliu S, Golomb D, Kleinfeld D, Ahissar E (2012) Dorsorostral snout muscles in the rat subserve coordinated movement for whisking and sniffing. Anat Rec (Hoboken) 295: 1181–1191. 22641389 10.1002/ar.22501 PMC4153473

[32] Haidarliu S, Simony E, Golomb D, Ahissar E (2010) Muscle architecture in the mystacial pad of the rat. Anat Rec (Hoboken) 293: 1192–1206. 20583263 10.1002/ar.21156

[33] Ahissar E, Ahissar M (1994) Plasticity in auditory cortical circuitry. CurrOpinNeurobiol 4: 580–587. 10.1016/0959-4388(94)90060-4 7812148

[34] Ahissar E, Abeles M, Ahissar M, Haidarliu S, Vaadia E (1998) Hebbian-like functional plasticity in the auditory cortex of the behaving monkey. Neuropharmacol 37: 633–655. 10.1016/s0028-3908(98)00068-9 9705003

[35] Ego-Stengel V, Shulz DE, Haidarliu S, Sosnik R, Ahissar E (2001) Acetylcholine-dependent induction and expression of functional plasticity in the barrel cortex of the adult rat. J Neurophysiol 86: 422–437. 11431522 10.1152/jn.2001.86.1.422

[36] Aertsen A, Vaadia E, Abeles M, Ahissar E, Bergman H, et al (1991) Neural interactions in the frontal cortex of a behaving monkey: signs of dependence on stimulus context and behavioral state. JHirnforsch 32: 735–743. 1821420

[37] Perkon I, Kosir A, Itskov PM, Tasic J, Diamond ME (2011) Unsupervised quantification of whisking and head movement in freely moving rodents. J Neurophysiol 105: 1950–1962. 21307326 10.1152/jn.00764.2010

[38] Knutsen PM, Pietr M, Ahissar E (2006) Haptic object localization in the vibrissal system: behavior and performance. J Neurosci 26: 8451–8464. 16914670 10.1523/JNEUROSCI.1516-06.2006 PMC6674338

[39] Hill DN, Curtis JC, Moore JD, Kleinfeld D (2011) Primary motor cortex reports efferent control of vibrissa motion on multiple timescales. Neuron 72: 344–356. 22017992 10.1016/j.neuron.2011.09.020 PMC3717360

[40] Moore JD, Deschenes M, Furuta T, Huber D, Smear MC, et al (2013) Hierarchy of orofacial rhythms revealed through whisking and breathing. Nature 497: 205–210. 23624373 10.1038/nature12076 PMC4159559

[41] Diamond ME, Heimendahl M, Arabzadeh E (2008) Whisker-Mediated Texture Discrimination. PLoS Biology 6: 1627–1630. 10.1371/journal.pbio.0060220 PMC2525693 18752356

[42] Li YQ, Takada M, Kaneko T, Mizuno N (1997) Distribution of GABAergic and Glycinergic Premotor Neurons Projecting to the Facial and Hypoglossal Nuclei in the Rat. The Journal of Comparative Neurology 378: 283–294. 9120066 10.1002/(sici)1096-9861(19970210)378:2<283::aid-cne10>3.0.co;2-t

[43] Szwed M, Bagdasarian K, Blumenfeld B, Barak O, Derdikman D, et al (2006) Responses of trigeminal ganglion neurons to the radial distance of contact during active vibrissal touch. J Neurophysiol 95: 791–802. 16207785 10.1152/jn.00571.2005

[44] Nguyen QT, Kleinfeld D (2005) Positive feedback in a brainstem tactile sensorimotor loop. Neuron 45: 447–457. 15694330 10.1016/j.neuron.2004.12.042

[45] Fisher J, Henzinger TA (2007) Executable cell biology. Nat Biotechnol 25: 1239–1249. 17989686 10.1038/nbt1356

[46] Solomon EP, Schmidt RR, Adragna PJ (1990) Human Anatomy & physiology: Saunders College Publishing.

[47] Ahissar E, Arieli A (2012) Seeing via miniature eye movements: A dynamic hypothesis for vision. Frontiers in Computational Neuroscience 6: 89. 23162458 10.3389/fncom.2012.00089 PMC3492788

[48] Kepecs A, Uchida N, Mainen ZF (2006) The sniff as a unit of olfactory processing. Chem Senses 31: 167–179. 16339265 10.1093/chemse/bjj016

[49] Bahar A, Dudai Y, Ahissar E (2004) Neural signature of taste familiarity in the gustatory cortex of the freely behaving rat. J Neurophysiol 92: 3298–3308. 15212421 10.1152/jn.00198.2004

[50] Wise SP, Jones EG (1977) Cells of origin and terminal distribution of descending projections of the rat somatic sensory cortex. J Comp Neurol 175: 129–157. 408380 10.1002/cne.901750202

[51] Killackey HP, Koralek KA, Chiaia NL, Rhodes RW (1989) Laminar and areal differences in the origin of the subcortical projection neurons of the rat somatosensory cortex. J Comp Neurol 282: 428–445. 2715391 10.1002/cne.902820309

[52] Woolston DC, La LondeJR, Gibson JM (1983) Corticofugal influences in the rat on responses of neurons in the trigeminal nucleus interpolaris to mechanical stimulation. Neuroscience Letters 36: 43–48. 6856202 10.1016/0304-3940(83)90483-4

[53] Lee SH, Carvell GE, Simons DJ (2008) Motor modulation of afferent somatosensory circuits. Nature Neuroscience 11: 1430–1438. 19011625 10.1038/nn.2227 PMC2597103

[54] Kleinfeld D, Ahissar E, Diamond ME (2006) Active sensation: insights from the rodent vibrissa sensorimotor system. Curr Opin Neurobiol 16: 435–444. 16837190 10.1016/j.conb.2006.06.009

[55] Xiao B, Oram T, Zlochiver V, Bagdasarian K, Ahissar E. Fast motor pathway from posteromedial thalamic nucleus to facial nucleus; 2012; New Orleans.

[56] Hemelt ME, Keller A (2008) Superior colliculus control of vibrissa movements. J Neurophysiol 100: 1245–1254. 18562549 10.1152/jn.90478.2008 PMC2544455

