# Supplementary figures and images for: Tactile Modulation of Whisking via the Brainstem Loop: Statechart Modeling and Experimental Validation
Source: PLoS One. 2013 Nov 27;8(11):e79831. doi: 10.1371/journal.pone.0079831 (PMC3842298; doi:10.1371/journal.pone.0079831)

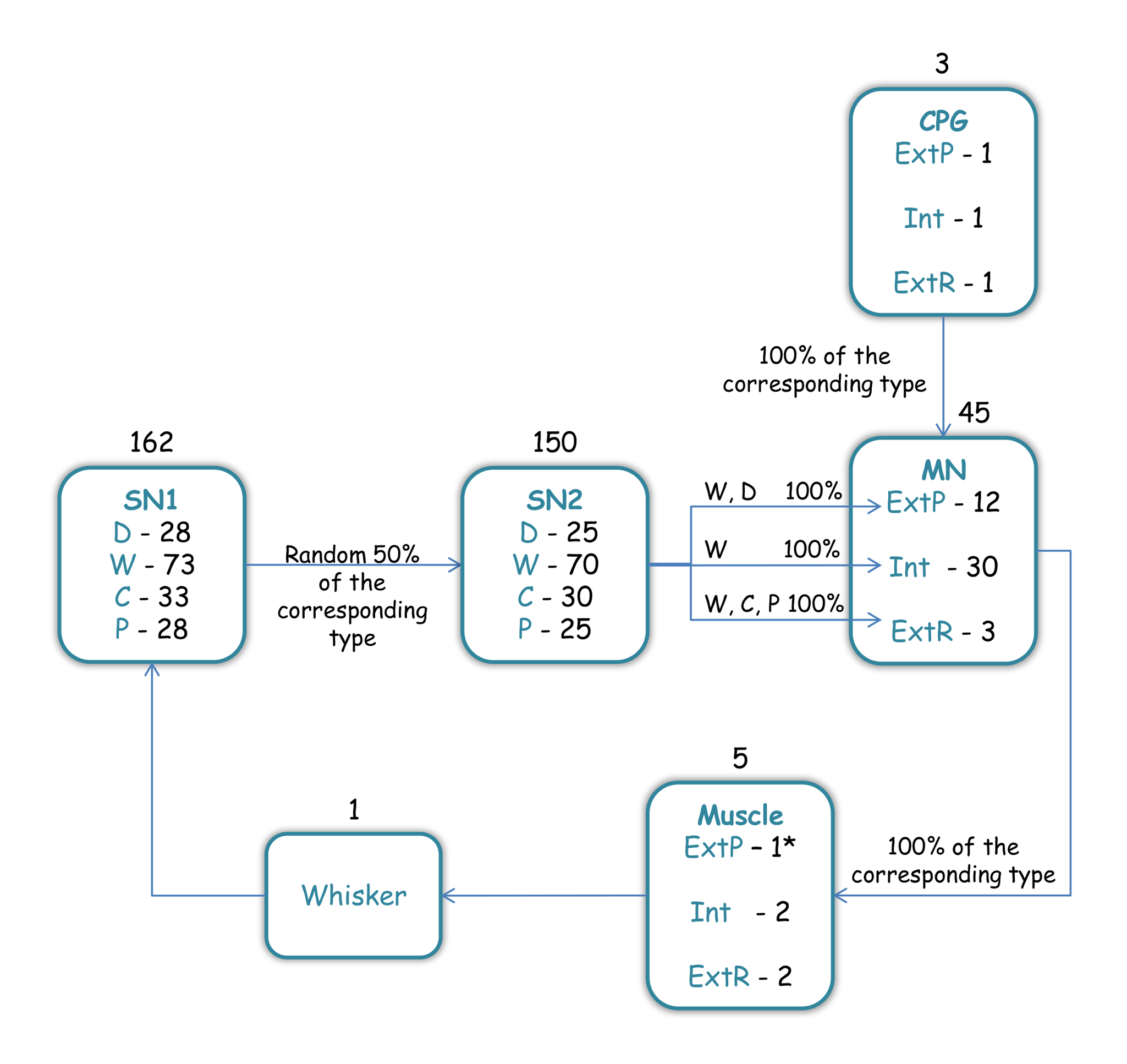

Supplement: Figure S1 — The number of elements that compose a single whisker's loop. Each whisker is innervated by a separated pool of primary afferents (SN1s), secondary afferents (SN2s) and motor efferents (MNs), which contain tens of neurons of several subtypes, as indicated in the scheme. For example, a single whisker is directly innervated by 162 SN1s which include 28 detach (D), 73 whisking (W), 33 contact (C), and 28 pressure (P) cells. Each type of SN1s innervates the corresponding type of SN2s, where a single SN2 is innervated by randomly chosen 50% SN1s of the corresponding type. Depending on the TIP-inducing configuration, different types of SN2s innervate different types of MNs (as indicated in figure 3B–G in the paper), with each MN innervated by all SN2s of the matched type. The “E-R” TIP-inducing configuration is displayed here. Each type of MNs innervates the corresponding type of muscle/s attached to the whisker (as indicated in figures 1B, 2B in the paper). In addition to this closed loop, all whiskers' MNs are innervated by the model CPGs, with each CPG innervating all MNs of the corresponding type. Note that no connections exist between sub-populations of neurons of a certain type (e.g., between whisking (SN1_W) and pressure (SN1_P) cells). * Two (instead of one) extrinsic protractor muscles are attached to the most rostral whisker in rows A–B. (TIF) [file pone.0079831.s001.tif]

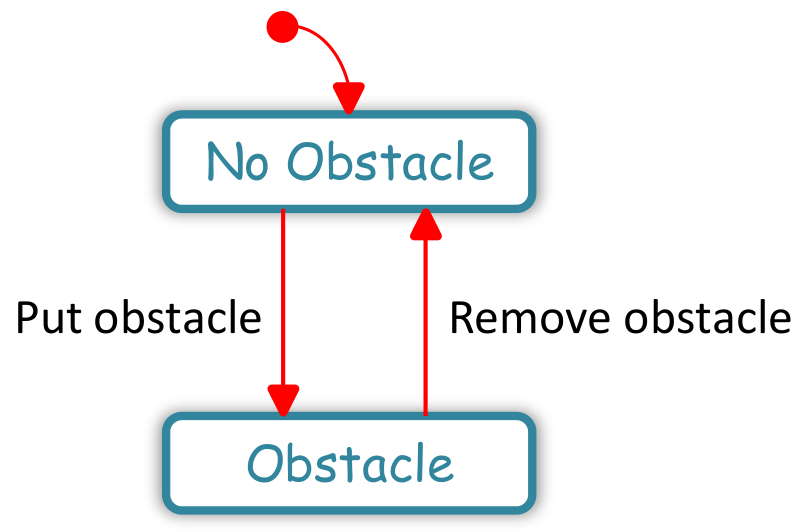

Supplement: Figure S2 — The statechart of the Obstacle element. The behavior of the Obstacle is described in File S1. (TIF) [file pone.0079831.s002.tif]
