# Supplementary material for: Tactile Modulation of Whisking via the Brainstem Loop: Statechart Modeling and Experimental Validation
Source: PLoS One. 2013 Nov 27;8(11):e79831. doi: 10.1371/journal.pone.0079831 (PMC3842298; doi:10.1371/journal.pone.0079831)
Supplement: File S1 — Supporting information. (DOCX) [file pone.0079831.s003.docx]

**Tactile modulation of whisking via the brainstem loop: Statechart modeling and experimental validation**

**Supporting information**

**Dana Sherman^1,2^, Tess Oram^1^, Dudi Deutsch^1^, Goren Gordon^1^, Ehud Ahissar*^1^ and David Harel*^2^**

^1^ Department of Neurobiology, Weizmann Institute of Science, Rehovot 76100, Israel

^2^ Department of Computer Science and Applied Mathematics, Weizmann Institute of Science, Rehovot 76100, Israel

*, equal contribution

**Model specifications**

**Number of elements in the model**

The brainstem loop model contained five rows of whiskers, with two rows of four whiskers and three rows of seven whiskers, corresponding to rows A-B and C-E in the rat. Each whisker was innervated by a separate pool of neurons that closed the loop between that whisker and the muscles attached to it, as shown in Figure S1.

**Model parameters**

The parameters in the model are listed below. Parameter values were set based on experimental data (indicated in the Source column). When empirical data were not available, a genetic algorithm (GA) was used to evaluate values (see Genetic algorithm). The algorithm converged into several optimal solutions that produced the desired free-air whisking and TIP motions. The values of the most stable solution are displayed below.

Whisker

| Parameter | Units | Value | Description | Source |
| --- | --- | --- | --- | --- |
| $\theta_{0}$ | deg | 70 | Angle at the beginning of simulation | [[1](#_ENREF_1)] |
| $\theta(t)$ | deg | $\theta\left( 0 \right)=\theta_{0}$ | Current angle |  |
| $\dot{\theta}_{0}$ | deg/sec | 0 | Angular velocity at the beginning of simulation |  |
| $\dot{\theta}(t)$ | deg/sec | $\dot{\theta}\left( 0 \right)=\dot{\theta}_{0}$ | Current angular velocity |  |
| $\dot{\theta}_{max}$ | deg/sec | 2000 | Maximal angular velocity | [[1](#_ENREF_1)] |
| *row* | index | 1-5 | Row number  (rows 1-5 correspond to rows A-E in the rat) |  |
| *index* | index | 1-7 | Index of column in the row  (up to 4 in four-whisker rows) |  |
| *xc* | m | 0.002*(*index* + 1) | Center of mass along the x-axis (caudal-rostral) | [[1](#_ENREF_1)] |
| *yc* | m | 0 | Center of mass along the y-axis (ventral-dorsal) | [[1](#_ENREF_1)] |
| *L* | m | 0.03 | Length |  |

Object

| Parameter | Units | Value | Description | Source |
| --- | --- | --- | --- | --- |
| *x* | m |  | Position along the x-axis (caudal-rostral) |  |
| *y* | m |  | Position along the y-axis (medial-radial) |  |
| *z* | m | 1-5  (index) | Position along the z-axis (ventral-dorsal).  The model assumes no whisker motion in this axis, thus z value simply indicates the row number in which the object is present |  |
| *present* | logical | True/False | True when object is present |  |

Whisking cells (SN1_W)

| Parameter | Units | value | Description | Source |
| --- | --- | --- | --- | --- |
| $P_{W}(\theta,\dot{\theta})$ | - | 0-1 | Firing probability: the current probability of the cell to fire.  $P_{W}(\theta,\dot{\theta})=K_{1}\cdot\frac{\theta_{range}-\vert\theta-\theta_{f}\vert}{\theta_{range}}+K_{2}\cdot\frac{\dot{\theta}}{\dot{\theta}_{max}}$  Set $P_{W}\left( \theta,\dot{\theta} \right)=0$: (1) when $P_{W}\left( \theta,\dot{\theta} \right)<0$, (2) upon cell’s firing |  |
| *threshold* | - | 0 | The threshold to be crossed in order to successfully fire ($P_{W}$ must be > 0) |  |
| *k1* | - | 0.84 | A relative-weight constant | GA |
| *k2* | - | 0.16 | A relative-weight constant; *k2*=1-*k1* | GA |
| *index* | index | 0-72 | Each of the whisker's 73 SN1_Ws takes a different index |  |
| $\theta_{f}$ | deg | 50 + *index* | Favorite angle: its whisker's angle to which the cell is most sensitive, i.e., fires with highest probability |  |
| $\theta_{range}$ | deg | 5.6 | The range of angles of its whisker to which the cell responds.  A cell responds to the range: $[\theta_{f}-\frac{\theta_{range}}{2},\theta_{f}+\frac{\theta_{range}}{2}]$ | GA |
| $t_{relay}$ | msec | 2 | Conductance time of the stimulus along the cell's axon to its post-synaptic target | [[2](#_ENREF_2)] |
| *ARP* | msec | 1 | Absolute refractory period duration | [[3](#_ENREF_3)] |
| *RRP* | msec | 3 | Relative refractory period duration | Na^+^ channels recovery time |

Contact (SN1_C)

| Parameter | Units | value | Description | Source |
| --- | --- | --- | --- | --- |
| $t_{response}$ | msec | 2-5 (2)  [range (median)] | Time to generate action potential in response to contact stimulus | [[4](#_ENREF_4)] |
| $t_{relay}$ | msec | 1 | Conductance time of the stimulus along the cell's axon to its post-synaptic target | [[2](#_ENREF_2)] |
| *ARP* | msec | 1 | Absolute refractory period duration | [[3](#_ENREF_3)] |
| *RRP* | msec | 3 | Relative refractory period duration | Na^+^ channels recovery time |
| *index* | index | 0-32 | Each of the whisker's 33 SN1_Cs takes a different index |  |

Pressure (SN1_P)

| Parameter | Units | value | Description | Source |
| --- | --- | --- | --- | --- |
| $t_{response}(x,y,L)$ | msec | 1-34 | Time to generate action potential in response to contact stimulus.  Changes for every cell according to the radial distance of contact between its whisker and an object.  $t_{response}=t_{delay}*F(x,y)$  $t_{delay}$ – "basic" delay of a cell; 8-34 (16) [range (median)].  F(x,y) – change in $t_{delay}$ as a function of object's radial distance, measured from whisker's base ($\frac{\sqrt{{(x-xc)}^{2}+{(y-yc)}^{2}}}{L}$). | [[4](#_ENREF_4),[5](#_ENREF_5)] |
| $t_{relay}$ | msec | 1 | Conductance time of the stimulus along the cell's axon to its post-synaptic target | [[2](#_ENREF_2)] |
| *ARP* | msec | 1 | Absolute refractory period duration | [[3](#_ENREF_3)] |
| *RRP* | msec | 3 | Relative refractory period duration | Na^+^ channels recovery time |
| *index* | index | 0-27 | Each of the whisker's 28 SN1_Ps takes a different index |  |

Detach (SN1_D)

| Parameter | Units | Value | Description | Source |
| --- | --- | --- | --- | --- |
| $t_{response}$ | msec | 1 | Time to generate action potential in response to detachment of its whisker from an object | [[4](#_ENREF_4)] |
| $t_{relay}$ | msec | 1 | Conductance time of the stimulus along the cell's axon to its post-synaptic target | [[2](#_ENREF_2)] |
| *ARP* | msec | 1 | Absolute refractory period duration | [[3](#_ENREF_3)] |
| *RRP* | msec | 3 | Relative refractory period duration | Na^+^ channels recovery time |
| *index* | index | 0-27 | Each of the whisker's 28 SN1_Ds takes a different index |  |

SN2 (W,C,P,D)

| Parameter | Units | Value | Description | Source |
| --- | --- | --- | --- | --- |
| *type* | - | W,C,P,D | Cell *type*: whisking (W), contact (C), pressure (P), or detach (D) | These SN2s also project to the thalamus, where the different types of information are relayed separately [[6](#_ENREF_6)]. |
| *index* | index | 0-T | Each of the whisker's SN2s of each *type* takes a different index: *T* = 69,29,24,24 for SN2 of *type* W,C,P,D |  |
| $t_{response}$ | msec | 1 | Time to generate action potential in response to stimuli from its pre-synaptic SN1s.  Not a parameter; results from simulation dynamics* | GA |
| $t_{relay}$ | msec | 1-4 | Conductance time of the stimulus along the cell's axon to its post-synaptic target |  |
| *ARP* | msec | 2 | Absolute refractory period duration | [[7](#_ENREF_7)] |
| *RRP* | msec | 3 | Relative refractory period duration | Na^+^ channels recovery time |

* The delay in firing of an SN2 following pre-synaptic stimuli ($t_{response}$) depended on the time it took the cell to cross a threshold. The time for crossing the threshold depended on the *threshold* (increased in the “RRP” state relative to “Rest”; value evaluated by GA), strength of the pre-synaptic stimuli and time interval between stimuli:

$$A_{SN2}\left( t \right)=A_{SN2}\left( t-1 \right)+\sum_{index=1}^{T} E_{SN1\_X_{index}}-C$$

where $t$ is the elapsed time from the moment the SN2 entered the “GenerateAP” state,

$A_{SN2}\left( t-1 \right)$ is the SN2 excitability value (arbitrary units) at time t-1,

$X$ is the *type* of information the SN2 relayed, i.e., whisking (W), contact (C), pressure (P) or detach (D),

$E_{SN1\_X_{index}}$ is the effect of a pre-synaptic cell "*index*" of the corresponding *type* ($X$), on its SN2 excitability,

$T$ is the number of primary afferents of *type* $X$ activated at time $t$,

$C$ is a constant.

Once an SN2 crossed *threshold*, its excitability values were zeroed ($t=0; A_{SN2}\left( 0 \right)=0$).

Thus the effect of an SN1 firing on its SN2 excitability depended on the synaptic strength ($E_{{SN1\_X}_{index}}$) and decreased with time, as moving away from the firing moment of the SN1 (embodied in $C\cdot(t-1)$).

$A_{SN2}\left( t \right)$ value was not changed if the SN2 was at the "absolute refractory period" state.

MN (ExtP, Int, ExtR)

| Parameter | Units | Value | Description | Source |
| --- | --- | --- | --- | --- |
| *type* | - | ExtP, Int, ExtR | MN *type*: extrinsic protractor, intrinsic, or extrinsic retractor | [[8](#_ENREF_8),[9](#_ENREF_9)] |
| $t_{response}$ | msec | 1-4 | Time to generate action potential in response to stimuli from its pre-synaptic sources (SN2s, CPG).  Not a parameter; results from simulation dynamics* | GA |
| $t_{relay}$ | msec | 1 | Conductance time of the stimulus along the cell's axon to its post-synaptic target | [[10](#_ENREF_10),[11](#_ENREF_11)] |
| *ARP* | msec | 2 | Absolute refractory period duration | [[10](#_ENREF_10)] |
| *RRP* | msec | 3 | Relative refractory period duration | Na^+^ channels recovery time |

* The delay in firing of an MN following pre-synaptic stimuli ($t_{response}$) depended on the time it took the cell to cross a threshold. The time for crossing the threshold depended on the *threshold* (increased in the “RRP” state relative to “Rest”; value evaluated by GA), strength of the pre-synaptic stimuli and time interval between stimuli:

$$A_{MN}\left( t \right)=A_{MN}\left( t-1 \right)+E_{CPG}+\sum_{index=1}^{T} E_{{SN2\_X}_{index}}-C$$

where $t$ is the elapsed time from the moment the MN entered the “GenerateAP” state,

$A_{MN}\left( t-1 \right)$ is the MN excitability value (arbitrary units) at time *t*-1,

$E_{CPG}$ is the effect of the pre-synaptic CPG on its MN excitability,

$X$ is the *type* of information the SN2 relayed, i.e., whisking (W), contact (C), pressure (P) or detach (D),

$E_{SN2\_X_{index}}$ is the effect of a pre-synaptic SN2_X number "*index"* on its MN excitability,

$T$ is the number of secondary afferents of *type* $X$ activated at time $t$,

$C$ is a constant.

Once an MN crossed threshold, its excitability values were zeroed ($t=0; A_{\mathrm{MN}}\left( 0 \right)=0$).

Thus the effect of a pre-synaptic source (CPG or SN2) firing on its MN excitability depended on the synaptic strength ($E_{CPG}$ or $E_{{SN2\_X}_{index}}$) and decreased with time, as moving away from the firing moment of the pre-synaptic source (embodied in $C\cdot(t-1)$).

$A_{MN}\left( t \right)$ value was not changed if the MN was at the "absolute refractory period" state.

Muscle (ExtP, Int, ExtR)

| Parameter | Units | Value | Description | Source |
| --- | --- | --- | --- | --- |
| *type* | - | ExtP, Int, ExtR | Muscle *type*: extrinsic protractor, intrinsic, or extrinsic retractor | [[12](#_ENREF_12)] |
| ${Ca}_{0}$ | M | 10^-7^ | Concentration of Ca^2+^ ions in muscle cytoplasm at rest | [[1](#_ENREF_1)] |
| $Ca(t)$ | M | $\frac{r_{0}\tau_{c}}{\tau_{c}-\tau_{r}}\left[ e^{\frac{-t}{\tau_{c}}}-e^{\frac{-t}{\tau_{r}}} \right]+Ca(t-1)e^{\frac{-t}{\tau_{c}}}$ | Concentration of Ca^2+^ ions released from the SR to the cytoplasm; $Ca\left( 0 \right)={Ca}_{0}$ | [[1](#_ENREF_1)] |
| $r_{0}$ | - | 2.55 | A constant | [[1](#_ENREF_1)] |
| $r(t)$ | - | $e^{\frac{-t}{\tau_{r}}}$ | The fraction of open ryanodine receptors (RyRs); $r\left( t \right)=r_{0}$ | [[1](#_ENREF_1)] |
| *t* | msec |  | Elapsed time following MN stimulation. Initialized to zero at stimulation onset time |  |
| $\tau_{c}$ | msec | 7.4 | Time constant of the decay of intracellular Ca^+2^ ions | [[1](#_ENREF_1)] |
| $\tau_{r}$ | msec | 5 | Decay time constant of *r* | [[1](#_ENREF_1)] |
| *A* | - | * | Force scaling factor | GA |
| *stimuliNum* | - | 0-(size of innervating MN pool) | Number of MN stimuli in the last 4 msec |  |
| *threshold* | index | $\frac{1}{3}$ of the innervating MN pool | Minimal number of *stimuliNum*, during a time-window of 4 msec, required for muscle contraction |  |

* Muscles force scaling factor:

| Muscle *type* | Value | Description |
| --- | --- | --- |
| Intrinsic | 0.30 |  |
| Pseudo intrinsic | 0.83 |  |
| Extrinsic protractor | $A=\left\{ \begin{aligned} 0.05, &\mathrm{CPG}-induced activation \\ 0.3, &sensory feedback-induced activation \end{aligned} \right.$ | i.e., muscle's ExtP_MNs triggerend by ExtP_CGP.  i.e., muscle's ExtP_MNs triggerend by SN2_D. |
| Extrinsic retractor | $A=\left\{ \begin{aligned} 0.05, &\mathrm{CPG}-induced activation \\ f(x), &sensory feedback-induced activation \end{aligned} \right.$ | i.e., ExtR_MNs triggerend by ExtR_CGP.  i.e., ExtR_MNs triggerend by SN2_C,P.  When activated by SN2_C,P, *A*=f(x), is a function of the activated number of SN2_C,P (x) in response to whisker-object contact.  f(x) ~ x (size principle of motor units recruitment); 0.3 ≤ f(x) ≤ 0.5 |

Once a muscle successfully crosses its *threshold*, its parameters’ updated values are passed to a Matlab function, which calculates muscle’s force and transforms it into whisker motion. The calculation beyond the scope of this study and is describes in detail in [[1](#_ENREF_1)]. Briefly, total muscle force, *F*, is composed of two components: $F=F_{c}+F_{l}$, where the $F_{c}$ component depends on $Ca(t)$ and *A* as follows: $F_{c}=A\frac{{Ca(t)}^{4}}{1+{Ca(t)}^{4}}$, and the $F_{l}$ component depends on muscle's length (See formulas (6)-(7) in [[1](#_ENREF_1)]).

CPG (ExtP, Int, ExtR)

| Parameter | Units | Value | Description | Source |
| --- | --- | --- | --- | --- |
| *type* | - | ExtP, Int, ExtR | CPG *type*: extrinsic protractor, intrinsic, or extrinsic retractor | [[13](#_ENREF_13)] |
| *cycleDuration* | msec | 4 | Time interval between successive stimuli of the corresponding type of MNs.  Corresponds to CPG’s firing rate. | GA |
| *currentCycle* | index | 0-cyclesNum | Current cycle’s number.  Only at initialization, *currentCycle* gets a non-positive value*:  0,-2,-21 for ExtP, Int, ExtR CPGs |  |
| *cyclesNum* | index | 10,17,18 | Number of successive stimuli by ExtP, Int, and ExtR CPGs.  Corresponds to “Activate” state’s duration** | [[13](#_ENREF_13)] |
| *silenceDuration* | msec | 110,82,78 | for the ExtP, Int, and ExtR CPG, respectively.  *silenceDuration* = 150 - *cycleDuration* * *cyclesNum*  (150 msec is whisk cycle duration, i.e., protraction + retraction) | [[13](#_ENREF_13)] |

* At the beginning of the simulation *currentCycle* gets a non-positive value, resulting in a delayed activation of the CPG (delayed by |*currentCycle*|**cycleDuration*, i.e., by 0,8,84 msec for the ExtP, Int, ExtR CPGs [[13](#_ENREF_13)]).

** Model execution results in the following periods of activity of the different CPGs throughout a whisking cycle:

| CPG *type* | Active period [msec] |
| --- | --- |
| ExtP | 0-40 |
| Int | 8-80 |
| ExtR | 84-150 |

**Muscle forces & whisker motion**

A major part of our model was based on another study done in Ahissar’s group, which constructed a transformation function that converts an MN’s spikes to whisker movement. The transformation is done in two parts: motor neurons spiking are first converted to intrinsic muscle force production, and muscle force is then translated into motion of the whiskers. Calculation in each part is composed of several steps, specified in great detail in [[1](#_ENREF_1)].

The transformation function was developed and written in a Matlab environment, and underwent several changes in order to fit our model.

The Matlab function is called many times during model execution (approximately every 1 msec), to recalculate the anticipated whiskers’ movement each time a motor neuron fires a spike. Function accuracy depends on whisking amplitude, since the viscoelastic tissue is modeled as a system of linear springs and dampers, an approximation appropriate for small protraction angles [[See Discussion in 1](#_ENREF_1)]. Thus, amplitudes <15° give very high accuracy while amplitudes >15° allow a less accurate calculation of whisker movement.

Original code is available at: <http://senselab.med.yale.edu/ModelDB/ShowModel.asp?model=127512>

Since the underlying code in our model is written in java, a java-Matlab linking tool was implemented, allowing to directly use the complex transformation function code that already exists in Matlab without converting it to Java code, an action that has a high probability of introducing bugs.

This tool, consisting of two parts – a server part and a client part, makes it possible to call Matlab functions from Java programs. The server can be run inside the Matlab installation on a network host. The client calls the server by using remote method invocation (RMI) and allows for calling Matlab function on the fly, without saving results to a temporary file.

Code for this tool is available at: <http://jamal.sourceforge.net/about.shtml>

**Genetic algorithm**

In order to find the most fitted values for parameters whose empirical data were not available, a genetic algorithm was used (a parametric analysis technique often used in [optimization](http://en.wikipedia.org/wiki/Optimization_%28mathematics%29) problems).

In genetic algorithms, a set of parameters, which encode [candidate solutions](http://en.wikipedia.org/wiki/Candidate_solution) (called individuals) to an optimization problem, evolves toward better solutions. The evolution starts from a population of randomly generated individuals and happens in generations. In each generation, the fitness of every individual in the population is evaluated, multiple individuals are [stochastically](http://en.wikipedia.org/wiki/Stochastics) selected from the current population (based on their fitness), and modified to form a new population. The new population is then used in the next iteration of the [algorithm](http://en.wikipedia.org/wiki/Algorithm). Commonly, the algorithm terminates when either a maximum number of generations has been produced, or a satisfactory fitness level has been reached for the population.

The parameters indicated by “GA” in the above tables were used. The algorithm was executed eight times, starting each time from 50 randomly generated individuals and converging to a satisfactory solution that accurately simulated whisker motion. Out of the eight solutions, four were found to be stable, where the stability of a solution is defined as follows: changing the value of each one of the evaluated parameters (while keeping the other parameters constant), still results in the desired motion. The larger the change that would still result in the optimal solution is, the more stable/robust the solution is. The most stable solution of the four stable solutions is presented here (for each parameter, a change of up to ±(5-10)% of its possible range still resulted in the desired motion).

**Model behavior in statecharts**

**Neurons**

Since the behavior of all types of neurons in the model is very similar, the behavior of a generic neuron is described. The statechart that defines the behavior of a generic neuron is displayed in paper Figure 3B and explained in the text. In the model, each type of neuron has its own statechart, which is very similar to the diagram in Figure 3B, but with two major differences between the different types of neurons:

1. Type of stimulus that triggers a neuron to fire:

| **Cell type** | **Firing depends on…** |
| --- | --- |
| SN1_W | Whisker’s angle and angular velocity |
| SN1_C | Contact time - upon touch |
| SN1_P | 1. Contact time - upon and during touch period 2. Radial distance of contact (stimulus strength) |
| SN1_D | Detachment time - upon detachment |
| SN2s, MNs | Strength of stimulus - must reach threshold |

The firing probability of the above cells is specified in detail in Model parameters.

1. Post-synaptic target:

| **Pre-synaptic cell** | **Post-synaptic target** |
| --- | --- |
| Primary afferents* | Secondary afferents* |
| Secondary afferents  CPG | Motor neurons |
| Motor neurons | Muscles |

* Each type of primary afferents (i.e., W, C, P or D) innervates SN2s of the corresponding type – that relay the same type of information

**CPG, Muscle and Whisker**

The statecharts of these elements are displayed in paper Figures 3A,C-D and are explained in the text.

**Obstacle**

The obstacle statechart has two states (Figure S2):

1. “No obstacle” – in which no obstacle is present.
2. “Obstacle” - in which an obstacle is present.

At the beginning of the simulation, no obstacle is present (*present* = “false”) and the obstacle is in the “No Obstacle” state. When a “Put obstacle” event is sent, the obstacle moves to the “Obstacle” state and an obstacle is added to the simulation at (*x*,*y*,*z*). The obstacle stays in this state until a “Remove obstacle” event is sent, setting *present* to “true”.

**Manager**

The Manager’s behavior is not described here, as it basically helps overcome technical issues, such as synchronization between instances. This component does not have any equivalent biological entity.

**References**

1. Simony E, Bagdasarian K, Herfst L, Brecht M, Ahissar E, et al. (2010) Temporal and spatial characteristics of vibrissa responses to motor commands. J Neurosci 30: 8935-8952.

2. Descheˆnes M, Timofeeva E, Lavalle´e P (2003) The Relay of High-Frequency Sensory Signals in the Whisker-to-Barreloid Pathway. The Journal of Neuroscience 23: 6778–6787.

3. Leiser SC, Moxon KA (2007) Responses of trigeminal ganglion neurons during natural whisking behaviors in the awake rat. Neuron 53: 117-133.

4. Szwed M, Bagdasarian K, Ahissar E (2003) Encoding of Vibrissal Active Touch. Neuron 40: 621–630.

5. Szwed M, Bagdasarian K, Blumenfeld B, Barak O, Derdikman D, et al. (2006) Responses of trigeminal ganglion neurons to the radial distance of contact during active vibrissal touch. J Neurophysiol 95: 791-802.

6. Yu C, Derdikman D, Haidarliu S, Ahissar E (2006) Parallel Thalamic Pathways for Whisking and Touch Signals in the Rat. PLoS Biology 4: 819-824.

7. Jacquin MF, Mooney RD, Rhoades RW (1986) Morphology, response properties, and collateral projections of trigeminothalamic neurons in brainstem subnucleus interpolaris of rat. Exp Brain Res 61: 457-468.

8. Klein BG, Rhoades RW (1985) Representation of Whisker Follicle Intrinsic Musculature in the Facial Motor Nucleus of the Rat. The Journal of Comparative Neurology 232: 55-69.

9. Herfst LJ, Brecht M (2008) Whisker movements evoked by stimulation of single motor neurons in the facial nucleus of the rat. J Neurophysiol 99: 2821-2832.

10. Martin MR, Biscoe TJ (1977) Physiological studies on facial reflexes in the rat. Quarterly Journal of Experimental Physiology 62: 209-221.

11. Yetiser S, Kahraman E, Satar B, Karahatay S, Akcam T Anatomy of the Extratemporal Facial Nerve in Rats. The Mediterranean Journal of Otology.

12. Haidarliu S, Simony E, Golomb D, Ahissar E (2010) Muscle architecture in the mystacial pad of the rat. Anat Rec (Hoboken) 293: 1192-1206.

13. Hill DN, Bermejo R, Zeigler HP, Kleinfeld D (2008) Biomechanics of the vibrissa motor plant in rat: rhythmic whisking consists of triphasic neuromuscular activity. J Neurosci 28: 3438-3455.
